# Anticancer Quinolinol Small Molecules Target Multiple Pathways to Promote Cell Death and Eliminate Melanoma Cells Resistant to BRAF Inhibitors

**DOI:** 10.3390/molecules30132696

**Published:** 2025-06-22

**Authors:** Xinjiang Wang, Rati Lama, Alexis D. Kelleher, Erika C. Rizzo, Samuel L. Galster, Chao Xue, Yali Zhang, Jianmin Wang, Jun Qu, Sherry R. Chemler

**Affiliations:** 1 Department of Pharmacology and Therapeutics, Roswell Park Comprehensive Cancer Center, Buffalo, NY 14263, USA; 2 Department of Chemistry, University at Buffalo, State University of New York, Buffalo, NY 14260, USA; 3 Department of Pharmaceutical Sciences, School of Pharmacy, University at Buffalo, State University of New York, Buffalo, NY 14260, USA; 4 Department of Biostatistics and Bioinformatics, Roswell Park Comprehensive Cancer Center, Buffalo, NY 14263, USA

**Keywords:** MMRi62, quinolinol, MDM2-MDM4, E3 ligase, cell death, targets, chemical robe

## Abstract

Small molecule inhibitors that target the E3 ligase activity of MDM2-MDM4 have been explored to inhibit the oncogenic activity of MDM2-MDM4 complex. MMRi62 is a small molecule that was identified using an MDM2-MDM4 E3 ligase-based high throughput screen and a cell-death-based secondary screen. Our previous studies showed that MMRi62 promotes MDM4 degradation in cells and induces p53-independent apoptosis in cancer cells. However, MMRi62 activity in solid tumor cells such as melanoma cells, especially in BRAF inhibitor resistant melanoma cells, have not been explored. Although its promotion of MDM4 degradation is clear, the direct MMRi62 targets in cells are unknown. In this report, we show that MMRi62 is a much more potent p53-independent apoptosis inducer than conventional MDM2 inhibitors in melanoma cells. A brief structure-activity study led to development of SC-62-1 with improved activity. SC-62-1 potently inhibits and eliminates clonogenic growth of melanoma cells that acquired resistance to BRAF inhibitors. We developed a pair of active and inactive SC-62-1 probes and profiled the cellular targets of SC-62-1 using a chemical biology approach coupled with IonStar/nano-LC/MS analysis. We found that SC-62-1 covalently binds to more than 15 hundred proteins in cells. Pathways analysis showed that SC-62-1 significantly altered several pathways including carbon metabolism, RNA metabolism, amino acid metabolism, translation and cellular response to stress. This study provides mechanistic insights into the mechanisms of action for MMRi62-like quinolinols. This study also suggests multi-targeting compounds like SC-62-1 might be useful for overcoming resistance to BRAF inhibitors for improved melanoma treatment.

## 1. Introduction

Small-molecule inhibitors targeting cancer-driver protein kinases and enzymes constitute a majority of the targeted therapies approved by FDA in the past decades [1]. These targeted therapeutics are designed to target the interfaces in ATP binding pockets, domains of kinase activation, catalytic domains of enzymes or ligand binding for inactivation of the kinase activity that drives the cancerous cell proliferation. Although significantly improved cancer treatment for many cancer types, these therapies without exception face three challenges: low responsive rate, short-lived efficacy and drug resistance. Low responsive rate may be caused by additional genetic alterations in addition to the alterations in the intended drug targets in different patients. Short-lived efficacy is often intertwined with drug resistance that includes intrinsic and acquired drug resistance. The intrinsic drug resistance can be mediated by tumor cell heterogeneity, differential expression of efflux pump, and tumor microenvironment, while acquired resistance of initial responsive cancer cells can be gained through distinct mechanisms including gene mutation and non-genomic alteration of the targeted signaling pathways [2]. For example, acquired resistance to first and second-generation EGFR inhibitors in lung cancer can be conferred by point mutations such EGFR T790M mutation and resistance to third-generation EGFR inhibitors can be conferred by EGFR C797S mutation [3]. In the case of mutant BRAF inhibitors in melanoma, the acquired resistance is conferred by mechanisms involving genetic and epigenetic changes that activate different signaling pathways such as MAPK to bypass the effect of BRAF inhibition [4]. Likewise, inhibitors targeting MDM2-p53 interaction for p53-based therapies face resistance mechanisms involving mutations in p53 and other components of the p53 pathway [5,6,7] including MDM4 overexpression [8,9,10].

Despite our understanding of the various resistance mechanisms, the root cause of the limited efficacy of targeted therapies is lack of potent cell killing effect. This is because most drug targets for targeted therapies are mutated or deregulated kinases or enzymes, whose inactivation most likely results in reduced proliferation rather than cell death. While reduced proliferation prolongs progression-free survival, it allows cancer cells to rewire to alternative pathways or the treatment pressure selects resistant cancer populations. Eventually, the once-shrank tumors adapt the blockade by the targeted therapies leading to regrowth of more aggressive subtypes. In case of targeting MDM2 for p53-based cancer therapy, drug response to MDM2 inhibitors may differ significantly in terms of whether apoptosis or growth arrest is the major endpoint. This is because p53 plays a differential effect in apoptosis and cell cycle arrest in different cell types, with mainly apoptosis in leukemic or lymphoma cells and mainly growth arrest in fibroblasts and other cell types [11]. Indeed, MDM2 inhibitors induced a mixture of apoptosis and growth arrest in cancer patients in clinical trials [12,13].

To identify inhibitors that target the oncogenic activity of MDM2 with better cell killing activity, we used a unique approach for screening the lead compound. Our strategy was to first identify small molecules that target the RING domain of MDM2 which carries the E3 ligase activity and then use apoptosis assay in the secondary screen of primary hits identified by the biochemical screening [14]. This strategy is based on findings that the RING domains of both MDM2 and MDM4 are essential for p53 regulation *in vivo* [15,16,17] and that the E3 ligase activity of MDM2-MDM4 RING heterodimers is required for both p53 regulation *in vivo* and p53-independent cell cycle regulation [18]. Using this strategy, we identified MMRi62 that modulates the E3 ligase activity of MDM2-MDM4, promotes MDM4 degradation in cells and potently induces p53-independent apoptosis in leukemic cells [19] and ferroptosis in pancreatic cells [20]. Although MMRi62 hits the intended targets of MDM2-MDM4 complex, it is not clear how MMRi62 potently induces cancer cell death in different death mode. In this study, we report that MMRi62 has advantages over conventional MDM2 inhibitors in cell death induction in melanoma cells and SC-62-1 as MMRi62 derivatives has improved activity and is useful in eliminating melanoma cells with acquired resistance. We also report the results of SC-62-1-targeted pathways using SC-62-1 chemical probes and quantitative proteomics.

## 2. Results and Discussion

### 2.1. MMRi62 Has Advantage of Preferential Induction of Apoptosis over MDM2-p53 Inhibitors in Melanoma Cells

**Figure 1:**
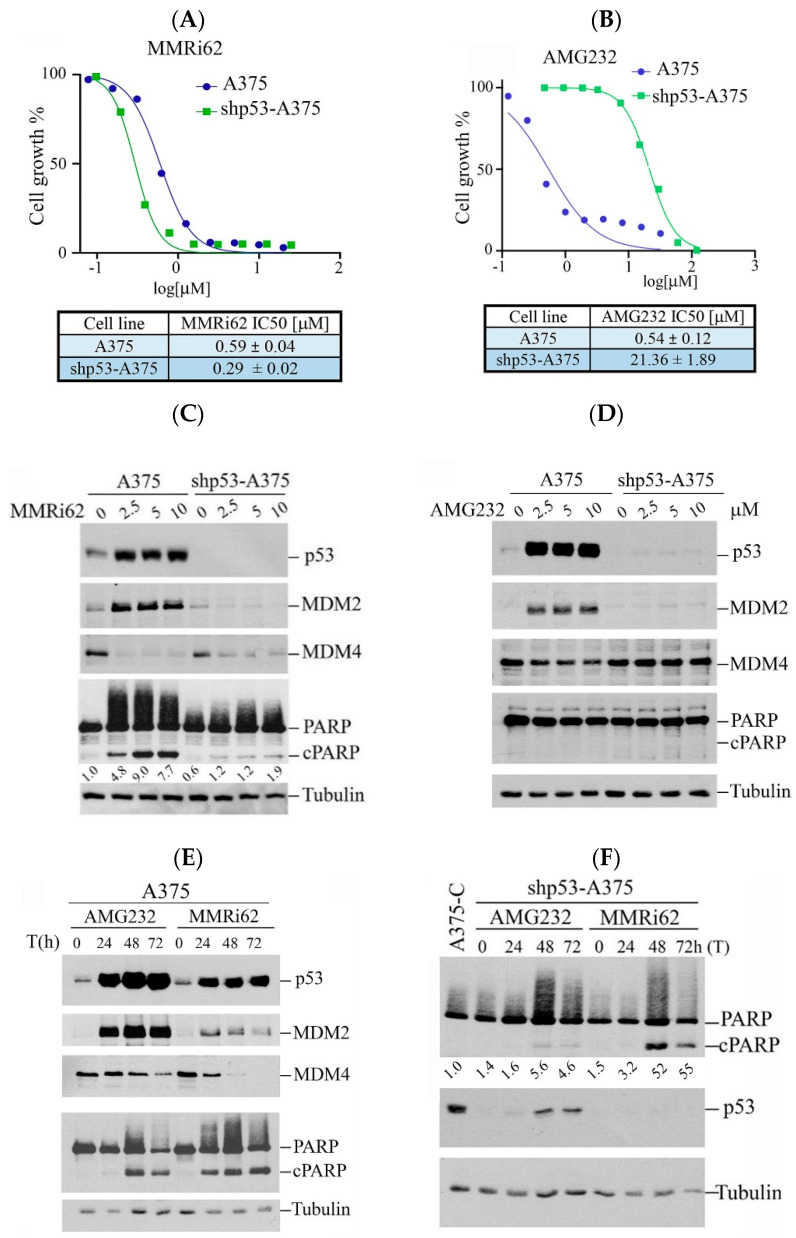
Westen Blot analysis of MMRi62 and AMG232 for the effect on MDM2, MDM4, p53 and cleaved PARP induction. (**A**,**B**) Dose response curves in the presence of MMRi62 (**A**) or AMG232 (**B**) for A375 and shp53-A375 cells. IC_50_s are shown at the bottom. The representative data of three independent experiments were shown and the IC_50_ values were obtained from three independent experiments. (**C**,**D**) WB analysis of MDM2, MDM4, p53 and cleaved PARP (cPARP) induction in A375 and shp53-A375 cells treated with MMRi62 (**C**) or AMG232 (**D**) for 24 h (**E**). WB analysis of MDM2, MDM4, p53 and cleaved PARP (cPARP) induction in A375 treated with AMG232 or MMRi62 for 48 h and 72 h (**F**), WB analysis of cleaved PARP (cPARP) and p53 in shp53A375 cells treated with AMG232 or MMRi62 for 48 h and 72 h. Tubulin is the protein loading control. The intensities of cPARP and tubulin bands in (**C**,**F**) were quantified by ImageJ1.54P software and fold increases in cPARP over untreated control shown under cPARP WB were obtained after normalization against tubulin.

Our previous study showed that MMRi62 induces MDM4 degradation and p53-independent apoptosis in leukemic cells [19]. We asked if this pro-apoptotic activity of MMRi62 also takes place in solid tumor cells such as melanoma cells. We used A375 and p53-knockdown shp53-A375 cells and treated them with either MMRi62 or AMG232, a potent MDM2 inhibitor that specifically disrupts MDM2-p53 interaction [21] as a comparison in anti-proliferation assays. Our results showed that MMRi62 and AMG232 had comparable IC_50_ values in A375 cells, but MMRi62’s anti-melanoma activity was not affected by p53 knockdown since A375 and shp53-A375 had similar IC_50_s (Figure 1A,B). In contrast to MMRi62, AMG232 showed a 40-fold reduced activity in shp53-A375 cells as compared to A375 cells (Figure 1B). These data demonstrated that the anticancer activity of AMG232 is p53-dependent and MMRi62’s activity is p53-independent, although both compounds induced p53 accumulation in A375 cells (Figure 1C,D). In contrast to AMG232, MMRi62-treated cells showed strong MDM4 downregulation in both A375 and shp53-A375 cells. MMRi62 treatment induced fast and strong apoptotic PARP cleavage (cPARP) in A375 cells at 24 h while AMG232 induced cPARP in A375 cells only after 48 h treatment (Figure 1C–E). MMRi62-induced cPARP was much weaker in shp53-A375 cells than in A375 cells, suggesting a major p53-dependent apoptosis mechanism was triggered in A375 cells. However, the MMRi62-induced p53-independent apoptosis was sustained up to 72 h in MMRi62-treated shp53-A375 cells (Figure 1C,F). Given that MMRi62 had similar IC_50_ values in A375 and shp53-A375 cells but with different levels of apoptosis, MMRi62’s anti-melanoma activity must involve multiple death modes including p53-dependent apoptosis, p53-independent apoptosis and non-apoptotic cell death.

### 2.2. SC-62-1, an MMRi62 Derivative with Improved Activity

**Figure 2:**
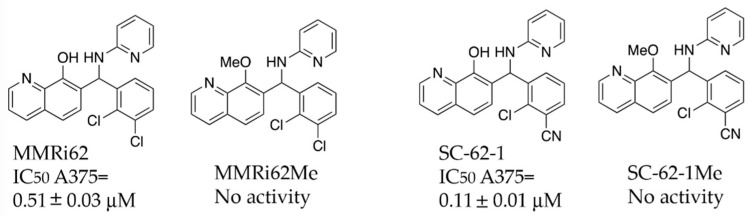
Chemical structure and antiproliferative activity of MMRi62, SC-62-1 and their inactive counterparts. Top: Chemical structures of MMRi62, MMRi62Me, SC-62-1 and SC-62-1Me. Bottom: The IC_50_ of MMRi62 and SC-62-1 in A375 cells.

A previous SAR study on MMRi62 and MMRi67 identified the critical role of the quinolinol’s hydroxyl moiety in anti-proliferation activity of these molecules [22]. Optimization of MMRi62 led to development of SC-62-1. We synthesized the methoxy counterparts of MMRi62 (MMRi62Me) and SC-62-1 (SC-62-1Me) and tested them together in A375 melanoma cells. Our results reached the same conclusion that hydroxyl group of MMRi62/SC-62-1 is essential for its activity since their methoxy counterparts lost activity (Figure 2). The calculated CLogP of SC-62-1 is 2.13, placing it in the realm of the mean value of ClogP for approved drugs, which has stayed consistently at 2.3–2.6 over a long period of time [23]. By replacing a chloro substituent with its bioisosteric nitrile [24] in the structure of SC-62-1, we have improved the water solubility over MMRi62 whose calculated CLogP is 3.42. To assess whether SC-62-1 is less toxic to non-cancer cells, we tested SC-62-1 in immortalized human keratinocytes HaCaT cells and obtained an IC_50_ of 0.61 ± 0.13 μM which is 5-fold higher than that of A375 cells, suggesting that SC-62-1 has limited degree of selectivity for melanoma cells over immortalized keratinocytes. We noticed that HaCaT cells grew as fast as A375 cells in culture which might suggest that SC-62-1 toxicity to HaCaT cells may be related to its cancer-like fast proliferation.

### 2.3. SC-62-1 Prevents Appearance of Drug Resistance and Effectively Kills Melanoma Cells with Acquired Resistance to BRAF Inhibitors

**Figure 3:**
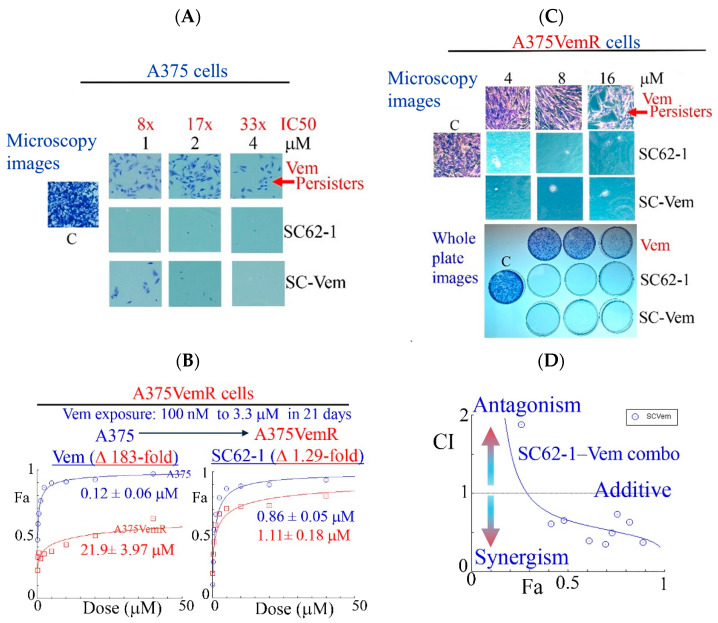
Effect of SC-62-1 and its combinations with Vemurafinib (Vem) on survival and proliferation of melanoma cells and Vem-resistant cells *in vitro*. (**A**) Microscopy images of crystal violet stained A375 cells after 2 week exposure to 1, 2, 4 μM Vem, SC-62-1 or their combinations. (**B**) Dose response curves of Vem-resistant A375 cells (A375VemR) with acquired resistance. (**C**) Microscopy images (top) and whole plates of crystal violet stained A375VemR cells after 8 day exposure to 4, 8, and 16 μM Vem, SC-62-1 or their combinations. (**D**) Combination index diagram obtained for SC-62-1-Vem combinations. CI < 1, synergism, CI = 1, additive, CI > 1, antagonism. Arrowheads indicate that the higher the CI values above 1, the more antagonistic they are, while the smaller the CI values below 1 toward zero, the more synergistic they are.

Like most kinase inhibitors, BRAF inhibitors (BRAFi) such as Vemurafinib (Vem) potently inhibit growth of BRAFV600E mutant melanoma cells but do not eliminate them by cell death. A substantial population of resistant cells or drug-resistant persisters survives the BRAFi treatment and lead to disease progression [25,26]. To know how SC-62-1 affects the drug-resistant persisters in melanoma, we performed experiments with continuous drug exposure of A375 cells for 2 weeks with either Vem or SC-62-1 in cell culture by replacing the medium with fresh drug-containing medium every 3 days. The drug-resistant persisters always existed in Vem-treated plates. Vem up to 4 μM (33× IC_50_ dose) could not eliminate the persister cells. In contrast, SC-62-1 at 1 μM eliminated persisters (Figure 3A, microscopy images of crystal violet stained cells, top vs. middle). SC-62-1-Vem combination at 1:1 ratio eliminated Vem-resistant persisters at 2 and 4 μM but failed at 1 μM, suggesting synergism at doses higher than 2 μM. We readily established Vem-resistant cell lines (A375VemR cells) by a stepwise increase in Vem concentration in cell culture. The A375VemR cells acquired Vem resistance by a ~180-fold increase in the IC_50_ value. However, the A375VemR cells showed only little resistance to SC-62-1 with just a ~1.3-fold IC_50_ change in the IC_50_ value compared to A375 cells (Figure 3B). Importantly, A375VemR cells remained sensitive to SC-62-1. Exposure of A375VemR cells to SC-62-1 or SC-62-1-Vem combo at >4 μM for 8 days completely wiped out these cells, but Vem alone at 4-to-16 μM failed to do so (Figure 3C). The results of the antiproliferation assay indicated that the SC-62-1-Vem combinations generated strong synergisms in the A375VemR cells in a wide range of drug concentrations with inhibition levels higher than 40% (Figure 3D). The combination reached strong synergism level at effect levels >90% inhibition with a CI < 0.3. These data suggest that SC-62-1 can prevent the development of resistance and eliminate the resistant to Vem treatment cells on its own or with Vem at doses higher than 2 μM.

### 2.4. Design, Synthesis and Chracterization of SC-62-1 Probes

The capability of MMRi62 and SC-62-1 in inducing cancer cell death made them attractive tool compounds for uncovering the molecular basis of cancer cell killing. To identify MMRi62/SC-62-1’s direct targets beyond the induced MDM4 degradation, we used an unbiased approach for profiling cellular targets with MMRi62/SC-62-1 probes coupled with quantitative proteomics.

**Figure 4:**
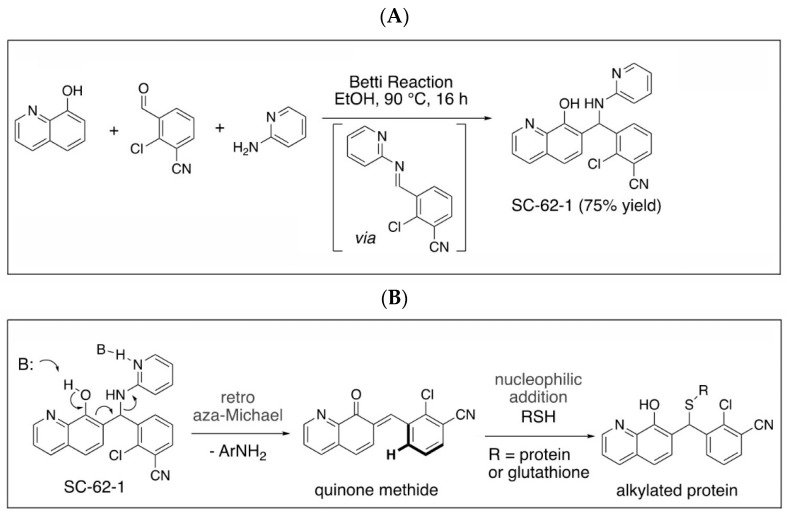

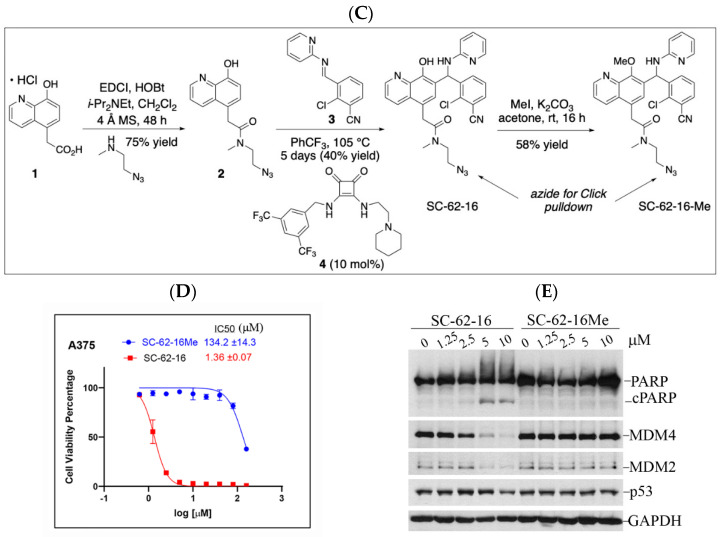
Synthesis of SC-62-1 and azide probes, hypothetical mechanisms of action of SC-62-1 and molecular characterization of azide probes. (**A**) Synthesis of quinolinol Betti base SC-62-1. (**B**) Hypothetical MOA for SC-62-1 as covalent inhibitors of target proteins via quinone methide to alkylate target proteins. (**C**) Synthesis routes for SC-62-16 and SC-62-16Me. (**D**) Anti-proliferation assays in A375 cells treated with SC-62-16 and SC-62-16Me for 72h. IC_50s_ values of the indicated compounds are shown. (**E**) Western blot analysis of SC-62-16 and SC-62-16Me effect on MDM4/MDM2 degradation and apoptotic PARP cleavage induction in treated A375 cells for 24 h. GAPDH is the protein loading control.

Quinolinol Betti bases like MMRi62/SC-62-1 are synthesized via a three component Betti reaction involving condensation of 8-hydroxyquinoline, an aromatic aldehyde and 2-aminopyridine (Figure 4A). We hypothesized that quinolinols act as covalent inhibitors as their primary mechanism of action (MOA) via a retro-aza-Michael-quinone-methide mechanism [27,28]. The reactive quinone methides target cysteine side chains of cellular proteins for covalent bonding which may alter the enzymatic activity or structural function of their targets (Figure 4B). The potential targets of quinone methides can be broad including key enzymes in various pathways required for cancer cell growth and survival whose inactivation should trigger cell death due to cellular stress in different cancer types. This establishes a rationale that the quinolinol Betti bases may compensate the limitations of kinase targeted therapies by targeting multiple pathways to trigger cell death as an endpoint. In fact, about 30% of the marketed drugs are covalent inhibitors including tamoxifen and other targeted therapies [29,30,31]. To identify targets for covalent binding of SC-62-1 predicted by our hypothetical MOA, we designed and synthesized the SC-62-16/SC-62-16Me pair of probes where SC-62-16 is the active compound and SC-62-16Me is the inactive counterpart (Figure 4C). This pair of probes used an alkyl azide on C(5) for a “Click” reaction to crosslink the probe with alkyne-functionalized beads for pulldown of probe-bound proteins [32]. As expected, SC-62-16 was active in A375 cells with an IC_50_ of 1.36 μM and promotes MDM4/MDM2 degradation while SC-62-16Me was inactive with an IC_50_ of 134 μM without inducing MDM4/MDM2 degradation (Figure 4D,E). Therefore, SC-62-16Me is a good negative control for identifying off-targets of SC-62-1 not involved in anticancer mechanisms. As expected, downregulation of MDM4 by SC-62-16 is accompanied with p53 accumulation up to 5 μM. However, both MDM4 and p53 were downregulated by 10 μM SC-62-16. We speculate that lower concentrations of SC-62-16 below 5 μM induced specific effect on MDM4-p53 axis while high concentrations of SC-62-16 at 10 μM or above caused inhibition of both MDM4 and p53 expression. A moderate degree of apoptotic PARP cleavage was induced by 5 μM and 10 μM of SC-62-16 without clear dose-dependency, suggesting that apoptosis is only a part of involved death mechanisms and higher concentrations of the compound tend to trigger non-apoptotic endpoints.

### 2.5. Identification of SC-62-16 Bound Proteins and Implicated Biological Pathways

**Figure 5:**
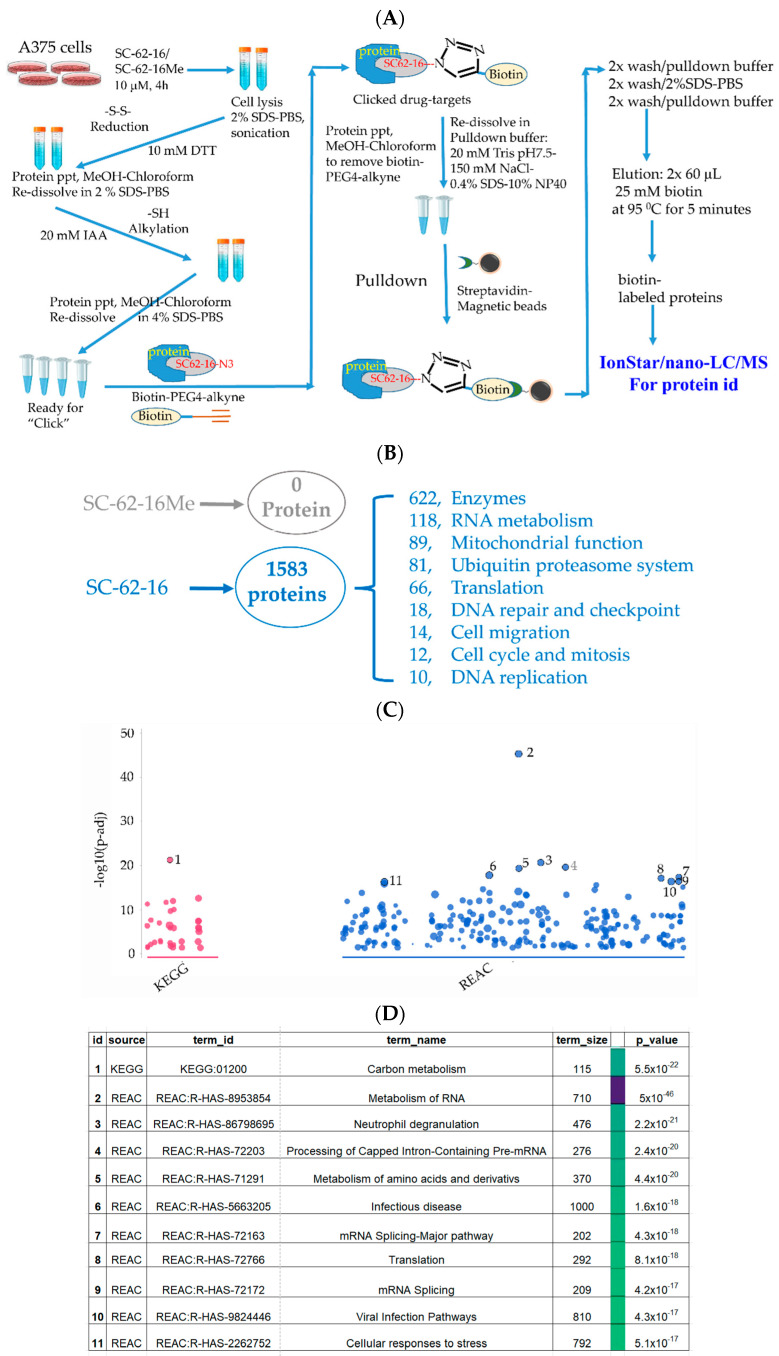
Identification of SC-62-16 bound proteins and targeted pathways. (**A**) Diagram for the optimized procedure of pulldown of SC-62-1-bound proteins. (**B**) Number of SC-62-16-bound proteins and their distributions in different categories. (**C**) Results of pathway analysis of the SC-62-16-bound proteins**.** The top 11 significantly altered pathways in using KEGG and REACTOME database are shown. (**D**) List of 11 pathways that are significantly impacted by SC-62-1.

We optimized a pulldown protocol in which biotin-PEG4-alkyne was used in click reaction [33,34] to tag a biotin moiety to SC-62-16/SC-62-16Me molecules that are covalently bound to proteins in treated cells, followed by pulldown of the biotin-tagged probe and its bound proteins by streptavidin-magnetic beads. The identities of proteins bound to the probes were then identified by quantitative IonStar/nano-LC/MS (Figure 5A). We treated A375 cells at 10 μM for 4 h before apoptosis occurs. Using 10 μM of the compounds ensured catching all possible SC-62-1 targets since this concentration induced strong MDM4 degradation and cell death at 24 h post-treatment (Figure 4E).

Our approach worked well since the negative control compound SC-62-16Me did not bring down any proteins while SC-62-16 brought down more than 1500 proteins (Figure 5B). More than 600 of the SC-62-16-bound proteins are enzymes. These proteins are classified in several biochemical/biological categories including RNA metabolism, mitochondrial function, ubiquitin proteasome system, protein translation, DNA repair and checkpoint, cell migration, cell cycle and mitosis and DNA replication. When the sequences of these proteins were subjected to pathway analysis using KEGG [35] and REACTOME database [36], it was revealed that the significantly altered pathways include those for carbon metabolism, RNA metabolism, amino acid metabolism, RNA splicing, protein translation and cellular stress response (Figure 5C,D).

When the SC-62-16-bound proteins were grouped by biological processes or functions in a common pathway and ranked by the protein numbers of each group, they could be classified into six groups as shown in Table 1. RNA metabolism was in the number one ranked category with a total of 124 proteins including 87 RNA binding proteins such as SUB1, FUS, EWSR1, RBMX, RRP43, DDX17, …etc. (Table 1), and 37 proteins in mRNA splicing including SF3B1, SF3A1, RBM39, SF3B4, PRPF8, U2AF1, …etc. (Table 1).

The second highest group impacted mitochondrial function, with a total of 94 proteins including 50 metabolic enzymes, namely, NDUFV1, MTHFD2, PYCR1, MCAT, GPD2, GSR, PPA2, …etc. (Table 1), and 44 proteins for mitochondrial tRNA metabolism and mitochondrial maintenance including VARS2, VARSL, MARS2, APEX1, HAP1, IARS2, WARS2, AFG3L2, MRM1, CARS2, REXO2, …etc. (Table 1).

The third group targets protein degradation, with a total of 55 proteins that include 18 ubiquitin E3 ligases, TRIM25, ARIH1, ARIH2, RNF20, CBL, HECTD1, HUWE1, TRIM18, RAD18, RBBP6, RNF113A, RNF149, RNF220, RNF34, RNFT2, TRIP12, UBR1, UBR4, 5 E2 ubiquitin-conjugating enzymes, UBE2D2, UBE2D3, UBE2L3, UBE2N, UBE2Z, 4 deubiquitinases, USP47, USP5, USP7, USP9 and 28 proteasome components including PSMD4, PSMB2, PSMB6, PSMB4, PSMB1, ECPAS, PSMA6, PSMA1, PSMB7, PSMD2, PSMA3, PSMD7, PSMD13, PSMA2, PSMC4, PSMC6, PSMD8, PSMB5, …etc. (Table 1).

The fourth group impacts protein translation, with a total of 50 proteins including 18 translation initiation factors including EIF3A, EIF6, EIF3G, EIF5A, EIF4G1, EIF5, …etc. (Table 1), 22 tRNA ligases including RARS1, VARS1, YARS1, AARS1, IARS1, EPRS1, TARS2, QARS1, …etc. (Table 1), and 10 ribosome proteins including GCN1, RRBP1, BMS1, NIP7, BOP1, MRTO4, LRRC59, GCN1, TMA7B, EEF1D. The fifth group contains 18 proteins involved in DNA repair and the DNA damage checkpoint response (RAD50, RAD23B, TREX1, MSH6, ERCC6L, RAD18, ERCC5, XRCC6, MRE11, APEX1, MMS19, BUB3, SMC2, MDC1, TP53BP1, FANCI, BRAT1, TOP1) and the last group is involved in cell migration and contains 12 proteins (AGRIN, RAC1, MIG8, NUDC, MIG10, MYH9, RHOC, GIT1, ARHGAP34, ARHGAP17, ARHGDIA, MYL6B) (Table 1).

Although our findings suggest that SC-62-1 alkylates ~1500 proteins in cells, the effect of alkylation on individual proteins is difficult to assess without further study of their effect on downstream components of their involved pathways. We speculate that the effect on the function of individual proteins can vary significantly and not all these potential alkylation events have negative impact on the function of target proteins. For example, there were 28 proteasome components among the targets, the collective effect of their inactivation on the proteasome activity could be significant and result in severe defect in proteasomal degradation of ubiquitinated proteins such as MDM4. However, MMRi62 and SC-62-16 but not SC-16Me induced fast proteasomal degradation of MDM4 as we showed in this study (Figure 1 and Figure 4E). These results suggested that the proteasome function was not defective at least for the induced proteasomal degradation of MDM4, despite that many of the proteasome components were alkylated by MMRi62 and SC-62-16. This suggests that while this target profile of SC-62-1 provides a comprehensive list of potential drug targets, only a limited number of protein targets have a meaningful consequence upon alkylation and are responsible for the drug effect. It appears that SC-62-1-mediated alkylation does not occur by random contact with SH-group since simply mixing SC-62-1 and glutathione in deuterated DMSOd_6_ and heating at 37 °C for 24 h did not produce any change in the ^1^H NMR spectrum of SC-62-1, which may indicate that alkylation occurs primarily inside proteins, possibly facilitated by acid/base catalysis and “intramolecularity”.

**Figure 6:**
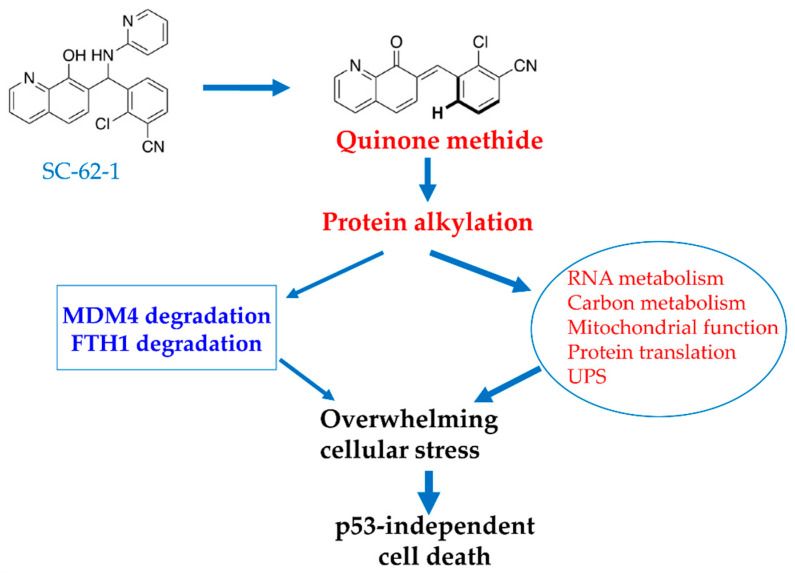
Proposed model for MOA of SC-62-1 in induction of cancer cell death. SC-62-1’s anticancer activity is via production of quinone methide which alkylates many target proteins. Its downstream effect includes induction of proteasomal degradation of MDM4 protein and lysosomal degradation of FTH1 and significant alteration of the pathways including RNA metabolism, carbon metabolism, mitochondrial function, protein translation and protein degradation. Collectively, induction of changes in these pathways by SC-62-1 creates overwhelming cellular stress that cannot be resolved, thus leading to p53-independent cell death.

A genetic screen for enriched genes in cells surviving SC-62-1 treatment might help sort out the targets that are responsible for the drug response [37]. Nevertheless, we speculate that SC-62-1 must have inactivated multiple key proteins and touched multiple pathways that prevent cells from overcoming the collateral damage. Based on the phenotypical effect of cell killing and the target profile of SC-62-1, we propose a working model as a possible mechanism of action for SC-62-1, shown in Figure 6. This model proposes that SC-62-1 acts as a multi-targeting alkylating agent. Through alkylating its target proteins, SC-62-1 not only induces proteasomal degradation of MDM4 and lysosomal degradation of FTH1, but also alters multiple pathways including RNA metabolism, mitochondrial function, protein translation and regulated protein degradation by proteasomes. The collective effect of its action creates overwhelming stressful state that cancer cells cannot overcome leading to p53-independent cell death in form of apoptosis in apoptosis-default cell types such leukemic cells or ferroptosis in ferroptosis-default cell types such as pancreatic and melanoma cells.

In summary, this study reports that quinolinol derivatives like MMRi62 and SC-62-1 preferentially induce cancer cell death compared to specific MDM2 inhibitors and BRAF inhibitors. They induce proteasomal degradation of MDM4 [19] and lysosomal degradation of FTH1 [20] but also target multiple other fundamental pathways for cell death induction. These compounds lack the specificity of conventional targeted therapies, a weakness measured by the concept of targeted therapies. However, pursuit of high specificity comes at the cost of short-lived efficacy and drug resistance, which is a well-established phenomenon for all targeted therapies. This limitation of targeted therapies lies in the capability of cancer cells easily bypassing the blockade of very specific drugs by mutating the well-defined interface or rewiring the affected signaling pathways. In fact, Mencher and Wang decades ago proposed that promiscuity of drugs can be a virtue compared to selective drugs [38]. Promiscuity has also been explored to overcome drug resistance to HIV-1 protease inhibitors [39]. In a recent study, 54 of 62 FDA-approved kinase inhibitors including Gleevec were reported to be promiscuous inhibitors with loose specificity of targeting at least two or more kinases. More interestingly, the study found that the more promiscuous type II inhibitors showed better pharmacodynamics than more specific type I inhibitors [40]. Therefore, it is not the specificity but the general toxicity and pharmacodynamics of a compound that determine its favorable therapeutic window. The toxicity of SC-62-1 is acceptable since its maximum tolerated dose in Scid mice is 60 mg/kg given every other day by intraperitoneal (I.P.) injection. We tested SC-62-1 for its *in vivo* efficacy in monotherapy in subcutaneous models of melanoma A375Luc in Scid mice at 57 mg/kg I.P. every other day and achieved ~50% reduction in tumor burden with body weight changes within 5%. However, due to small mouse number and large individual variation in tumor size, the results did not reach statistical significance. Future studies with a larger number of mice and development of derivatives with improved cancer cell selectivity will inform the potential of these quinolinol compounds as a new class of anticancer agents.

## 3. Materials and Methods

### 3.1. Representative Chemistry Methods

**(±)-2-Chloro-3-((8-hydroxyquinolin-7-yl)(pyridin-2-ylamino)methyl) benzonitrile (SC-62-1)**

To a 250 mL round bottomed flask equipped with a magnetic stir bar, 2-chloro-3-cyanobenzaldehyde (1.00 g, 6.04 mmol) was added along with 2-aminopyridine (568 mg, 6.04 mmol). 50 mL of absolute ethanol was added to the flask and the mixture was stirred until solids fully dissolved, at which point 8-hydroxyquinoline (1.05 g, 7.25 mmol) was added. The reaction flask was heated to 90 °C and refluxed for 24 h during which an off-white solid precipitated. The solution was allowed to cool to room temperature and to stand for several hours, then the resulting solid was isolated by filtration, giving **SC-62-1** (1.75 g, 75% yield) as an off-white powder. Mp = 190–192 °C; ^1^H NMR (300 MHz, CDCl_3_) δ 8.77 (d, *J* = 3.0 Hz, 1H), 8.13 (d, *J* = 7.2 Hz, 1H), 8.09 (d, *J* = 4.1 Hz, 1H), 7.97 (d, *J* = 7.9 Hz, 1H), 7.59 (d, *J* = 6.4 Hz, 1H), 7.50–7.26 (m, 6H), 6.70 (d, *J* = 6.4 Hz, 1H), 6.66–6.57 (m, 1H), 6.36 (d, *J* = 8.4 Hz, 1H), 5.55 (d, *J* = 6.2 Hz, 1H); ^13^C NMR (75 MHz, CDCl_3_) δ 157.2, 149.8, 148.3, 141.5, 138.2, 137.7, 136.1, 136.0, 133.0, 132.9, 127.9, 127.4, 127.0, 122.2, 120.6, 117.9, 116.2, 114.3, 114.0, 107.1, 53.6; IR (neat film): 3346, 2923, 2233, 1600, 1571, 1517, 1501 cm^−1^; HRMS (ESI) calculated for C_22_H_16_ClN_4_O [M + H]^+^: 387.1016, found 387.1007.

**(±)-*N*-(2-azidoethyl)-2-(7-((2-chloro-3-cyanophenyl)(pyridin-2-ylamino)methyl)-8-hydroxyquinolin-5-yl)-*N*-methylacetamide (SC-62-16)**

(Note: The 3-component Betti reaction above did not work for this 5-substituted-8-hydroxy quinoline, it is much less reactive than 8-hydroxy quinoline). To a 10 mL reaction tube, the imine **3** (42 mg, 0.18 mmol) was added, followed by the amide **2** (40 mg, 0.14 mmol, 0.8 eq.) dissolved in PhCF_3_ (0.5 mL). The squaramide catalyst **3** (8 mg, 0.039 mmol, 0.1 eq.) was also added to the reaction tube. Flame-dried 4 Å molecular sieves (30 mg) were added, and the reaction tube was sealed and kept in a dark environment at 105 °C for 5 days. The reaction mixture was cooled to rt, diluted with CH_2_Cl_2_, and filtered through Celite then concentrated to a crude mixture. The crude was then recrystallized from MeOH to yield 42 mg of **SC-62-16** as light brown crystals in 40% yield. The NMR data indicates the presence of amide rotamers due to hindered rotation. Mp = 162–164 °C; ^1^H NMR (400 MHz, CDCl_3_) δ 8.79 (dd, *J* = 4.2, 1.5 Hz, 1H), 8.36 (ddd, *J* = 14.3, 8.6, 1.5 Hz, 1H), 8.08 (ddd, *J* = 5.0, 1.9, 0.8 Hz, 1H), 7.94 (dt, *J* = 7.9, 2.5 Hz, 1H), 7.59 (dd, *J* = 7.7, 1.6 Hz, 1H), 7.49 (ddd, *J* = 8.6, 4.2, 1.6 Hz, 1H), 7.45–7.30 (m, 2H), 7.25 (s, 1H), 6.69 (dd, *J* = 6.5, 2.4 Hz, 1H), 6.61 (ddd, *J* = 7.2, 5.0, 0.9 Hz, 1H), 6.38 (d, *J* = 8.4 Hz, 1H), 5.54 (d, *J* = 6.5 Hz, 1H), 4.08–3.91 (m, 2H), 3.51 (t, *J* = 5.4 Hz, 2H), 3.47–3.33 (m, 3H), 3.07 (s, 2H), 2.92 (s, 1H); ^13^C NMR (101 MHz, CDCl_3_) δ 170.5, 157.8, 149.3, 148.0, 147.8, 142.9, 138.5, 136.8, 135.4, 133.7, 133.4, 132.8, 127.9, 127.5, 122.5, 121.7, 120.8, 115.9, 113.8, 113.0, 108.8, 51.8, 51.7, 49.2, 48.7, 48.5, 47.0, 37.2, 36.7, 35.8, 32.5; HRMS (ESI) calculated for C_27_H_24_ClN_8_O_2_ [M + H]^+^: 527.1711, found 527.1704.

The full synthesis and characterization details of all new compounds are provided in the Supplemental Information (SI).

### 3.2. Biological Assays and Methods

#### 3.2.1. Cell Culture

Melanoma cell line A375 was cultured in DMEM-10% fetal bovine serum, 50 U/mL penicillin and 50 μg/mL streptomycin. Cell line with stable p53 knockdown shp53-A375 was established using pLKO.1-p53 (purchased from Addgene) (Plasmid #19119) [41] followed by puromycin selection at 1 μg/mL for 2 days, then clonal expansion in puromycin-free medium.

#### 3.2.2. Western Blotting Analysis and Crystal Violet Staining

The Western blotting procedure and the relevant antibodies was described previously [19]. For crystal violet staining, cell plates were fixed with 4% (*w*/*v*) paraformaldehyde for 15 min followed by 30 min of staining in 0.1% (*w*/*v*) crystal violet solution in 20% (*v*/*v*) Ethanol-80% (*v*/*v*) PBS. After rinsing with tap water, the plates were airdried and the images were taken on Olympus IX73 inverted fluorescence microscope at a magnitude of 20×. The images of whole plates were taken on iPhone.

#### 3.2.3. IC_50_ Measurement and Analysis

The procedure for measuring IC_50_ was described previously [19]. Some data were obtained by Chou-Median-Effect Equation using CompuSynversion 1.0 (CompuSyn.exe) software [42] and some were obtained by GraphPad (Prism 8) using affected fractions of compound-treated wells normalized against no-drug control wells with non-linear regression model.

#### 3.2.4. Affinity Pulldown and Identification of SC-62-16/SC-62-16Me Covalently Bound Proteins

The procedure uses 2% (*w*/*v*) SDS-PBS lysis buffer to lyse the treated cells followed by treatment with 10 mM DTT, at 70 °C for 15 min to dissociate all non-covalent protein–protein interaction and reduce all the disulfide bonds among peptides. Then, the proteins were precipitated with 100% (*v*/*v*) methanol and 20% (*v*/*v*) chloroform, then were re-dissolved in 2% (*w*/*v*) SDS-PBS buffer by sonication. Before the “click” reaction, the samples were treated with iodoacetamide to alkylate sulfhydryl groups from further forming disulfide bonds. After “click” reaction with Biotin-PEG4-alkyne, the biotin-tagged compound-bound proteins were pulled down with magnetic Streptavidin beads. The bead-bound proteins were then eluted by 25 mM biotin heated at 95 °C for 5 min.

The click reaction conditions to link Biotin-PEG4-alkyne to SC-62-16 and SC62-16Me azide were adapted from a report by A. J. Pradhan et al. [43]. The conditions for biotin-streptavidin lysate preparation and biotin-streptavidin affinity purification and elution of compound-bound proteins were adapted from the method reported by J. S. Cheah et al. [44]. Biotin-PEG4-alkyne was purchased from Sigma-Aldrich (St. Louis, MO, USA) (cat# 764213-5MG) and PuroMAG™ Magnetic Beads-Streptavidin were purchased from Luna Nanotech (Markham, ON, Canada, Cat# MGB-STRP-10). Briefly, A375 cells (20 × 10 cm plates at 90% confluency) were treated with 10 μM SC-62-16 or SC-62-16Me for 4 h. After collecting the cells in a 50 mL centrifuge tube, they were washed with PBS and resuspended in 25 mL PBS and added 20% (*w*/*v*) SDS to final 2% (*w*/*v*) SDS-PBS followed by 20 cycles of sonication (30”-on-30”-off at 30% intensity of output) (Fisher Scientific Sonic Dismembrator Model 100 from Fisher Scientific, Waltham, MA, USA). Then, the cell lysate was treated with 10 mM DTT at 70 °C for 15 min. After centrifugation of the lysate at 50,000× *g* at 20 °C for 2 h, the soluble proteins were precipitated with 1× volume of methanol and 1/5× volume of chloroform with 5 min vigorous vortexing and 10 min centrifugation. After re-dissolving the proteins in 2% (*w*/*v*) SDS-PBS aided with sonication, the soluble protein solution was treated with 20 mM iodoacetamide for 30 min in darkness. Then, the proteins were precipitated again with methanol-chloroform and washed with cold methanol and resuspended in 4% (*w*/*v*) SDS-PBS. The 10× click reaction stock reagents were then added, to make a final concentration of 1 mM CuSO_4_, 1 mM TCEP, 0.25 mM TBTA and 0.25 mM Biotin-PEG4-alkyne. After a brief vortex, the mixture was incubated at 37 °C for 1 h. Then, the proteins were precipitated with methanol-chloroform followed by two washes with methanol with sonication. The proteins were re-dissolved in 3 mL of biotin-streptavidin lysis buffer (50 mM Tris HCl pH 7.4, 150 mM NaCl, 0.4% (*w*/*v*) SDS, 1% (*v*/*v*) NP, 1 mM EGTA and 1.5 mM MgCl_2_) and underwent 10 cycles of 30 secs-on-30 secs-off sonication in Sonic Dismembrator followed by centrifugation at maximal speed in a microcentrifuge to remove insoluble proteins. Then, the biotin-labeled proteins were pulled down with PuroMAG™ Magnetic Beads-Streptavidin at 100 μL of flurry beads/1 mL protein solution, rotate the sample tubes overnight at 4 °C. Then, the beads were washed twice with 1 mL of biotin-streptavidin lysis buffer with vigorous vortexing for 5 min followed by two washes with 2% SDS-PBS and then two washes with biotin-streptavidin lysis buffer. The proteins were eluted with two elution with 60 μL of 20 mM biotin in 50 mM Tris pH 8.3 and the identity of proteins were determined by high-quality and robust protein quantification by IonStar/nano-LC/MS at proteomics core facility run by Dr. Jun Qu [45] at the New York State Center of Excellence Bioinformatics and Life Sciences (CBLS), University at Buffalo.

#### 3.2.5. Pathway Analysis of SC-62-16/SC-62-16Me Covalently Bound Protein

Genes encoding the proteins identified by mass spectrometry in SC-62-16 samples were used to run pathway enrichment analysis using g:Profiler [46] and functional annotation using DAVID [47,48]. For the pathway enrichment runs, KEGG [35] and Reactome database [36] were used for the pathway search. The pathways with Benjamini–Hochberg adjusted *p*-value < 0.05 were ranked by their *p* values with the lowest as the top hit.

## Figures and Tables

**Table 1:** SC-62-16 covalent binding proteins.

Pathway Ranking	Pathway Name	Proteins Number	PROTEIN Names (Some Are Partial List)
1	RNA metabolism	124	87 RNA binding proteins such as SUB1, FUS, EWSR1, RBMX, RRP43, DDX17,
			DDX9, ADAR, SRB, RNH1, LUC7L2, ILF3, UBAP2L, SERBP1, KHDRBS1, NONO,
			IGF2BP2, SRRT, FTSJ3, MED23, RBM4, RCL1, EXOSC3, SYNCRIP, DHX30, EBP2,
			REXO2, ZFR, DDX1, …
			37 mRNA splicing such as SF3B1, SF3A1, RBM39, SF3B4, PRPF8, U2AF1, SCAF11,
			SF1, SFRS19, SRSF9, SRSF4, SFRS18, SF4, SFRS14, SFPQ, SRSF1, SRSF2, SRSF7,
			SRSF3, SPF45, SAP145, PRPF4, SRRM2, …
2	Mitochondrial function	94	50 metabolic enzymes such as NDUFV1, MTHFD2, PYCR1, MCAT, GPD2, GSR,
			PPA2, PC, PCCA, MDH2, SHMT2, GOT2, ECHS1, IDH3A, CS, MCCC1, UQCRC1,
			SUCLG1, ALDH2, ECH1, HAGH, FH, IVD, ACAT1, ACADVL, DBT,
			SUCLG2, PDHB, CPOX, DLAT, AK2, PDHA1, COQ7, PDHX, HIBADH,
			SDHB, PRDX3, ATP5F1A, UQCRC2, ATP5F1B, NDUFS1, PGAM5,
			ATP5PB, HSD17B10, HADHB, GLS, ATP5PO, DLST, MECR, OGDH,….
			10 for mitochondrial tRNA metabolism: VARS2, VARSL, MARS2, IARS2,
			MRM1, CARS2, REXO2, TARS2, RPUSD3, WARS2.
			34 proteins for mitochondrial maintenance: APEX1, HAP1, WARS2, AFG3L2,
			TOMM70, PMPCB, CLPB, HSPD1, HSPA9, SLC25A24, SCAMC1, MTCH2,
			TUFM, ETFA, LRPPRC, SLC25A3, SSBP1, SLC25A22, HSPE1, DAP3, VDAC2,
			VDAC1, MRPS27, CCDC51, MTX1, MRPS5, TMEM126B, TMEM11, IMMT,
			MINOS2, LETM1, MAVS, CYCS, DNM1L.
3	Protein degradation	55	18 ubiquitin E3 ligases:
			TRIM25, ARIH1, ARIH2, RNF20, CBL, HECTD1, HUWE1, TRIM18, RAD18,
			RBBP6, RNF113A, RNF149, RNF220, RNF34, RNFT2, TRIP12, UBR1, UBR4
			5 ubiquitin-conjugating enzymes: UBE2D2, UBE2D3, UBE2L3, UBE2N, UBE2Z,
			4 deubiquitinases: USP47, USP5, USP7, USP9
			28 proteasome components: PSMD4, PSMB2, PSMB6, PSMB4, PSMB1, ECPAS,
			PSMA6, PSMA1, PSMB7, PSMD2, PSMA3, PSMD7, PSMD13, PSMA2, PSMC4,
			PSMC6, PSMD8, PSMB5, PSMA5, PSMD11, PSMC3, PSMD1, PSMC1, PSMD4,
			PSMA7, PSMC2, PSMA4, PSMD12.
4	Protein translation	50	18 translation initiation factors: EIF3A, EIF6, EIF3G, EIF5A, EIF4G1, EIF5, EIF3I,
			EIF2S2, CDC123, EIF5B, EIF2S3, EIF3D, EIF3H, EIF3B, EIF3K, EIF4G3, EIF4G2,
			EIF4B.
			22 tRNA ligases, RARS1, VARS1, YARS1, AARS1, IARS1, EPRS1, TARS2,
			QARS1, HARS1, MARS1, WARS2, FARSB, IARS2, MARS2, AARS2, TARS1,
			SARS1, CARS1, NARS1, GARS1, WARS1, FARSA.
			10 ribosome proteins: GCN1, RRBP1, BMS1, NIP7, BOP1, MRTO4, LRRC59,
			GCN1, TMA7B, EEF1D.
5	DNA repair and	18	RAD50, RAD23B, TREX1, MSH6, ERCC6L, RAD18, ERCC5, XRCC6, MRE11,
	checkpoint response		APEX1, MMS19, BUB3, SMC2, MDC1, TP53BP1, FANCI, BRAT1, TOP1.
6	Cell migration	12	AGRIN, RAC1, MIG8, NUDC, MIG10, MYH9, RHOC, GIT1, ARHGAP34,
			ARHGAP17, ARHGDIA, MYL6B.

## Data Availability

All data are included in the article and Supplementary Materials.

## Supplementary Materials

The following supporting information can be downloaded at: https://www.mdpi.com/article/10.3390/molecules30132696/s1, References [49,50,51,52] are cited in the supplementary materials.
