# Supplementary material for: Anticancer Quinolinol Small Molecules Target Multiple Pathways to Promote Cell Death and Eliminate Melanoma Cells Resistant to BRAF Inhibitors
Source: Molecules. 2025 Jun 22;30(13):2696. doi: 10.3390/molecules30132696 (PMC12251381; doi:10.3390/molecules30132696)
Supplement: Supplementary file 1 [file molecules-30-02696-s001.zip › molecules-3657041-supplementary.pdf]

# Anticancer quinolinol small molecules target multiple pathways to promote cell death and eliminate melanoma cells resistant to BRAF inhibitors

Xinjiang Wang [1]*, Rati Lama [1], Alexis D. Kelleher [2], Erika C. Rizzo[2], Samuel L. Galster[2], Chao Xue [3], Yali Zhang [4], Jianmin Wang [4], Jun Qu [3] and Sherry R. Chemler[2]*

[1]Department of Pharmacology and Therapeutics, Roswell Park Comprehensive Cancer Center, Buffalo, NY 14263, USA

[2]Department of Chemistry, University at Buffalo, State University of New York, Buffalo, NY 14260, USA

[3]Department of Pharmaceutical Sciences, School of Pharmacy, University at Buffalo, State University of New York, Buffalo, NY 14260, USA

[4]Department of Biostatistics and Bioinformatics, Roswell Park Comprehensive Cancer Center, Buffalo, NY 14263, USA

*Correspondence: schemler@buffalo.edu; xinjiang.wang@roswellpark.org

## Supporting Information

### General Experimental Information

All reagents were used out of the bottle as purchased from the supplier without further purification unless otherwise noted. $^{1}$H NMR spectra were recorded in $CDCl_3$ (using 7.26 ppm for reference from residual $CHCl_3$), DMSO-$d_6$ (using 2.50 ppm for reference from residual DMSO), acetone-$d_6$ (using 2.06 ppm for reference from residual acetone) at 300, 400 or 500 MHz. $^{13}$C NMR spectra were recorded in $CDCl_3$ (using 77.0 ppm as internal reference), DMSO-$d_6$ (using 40.0 ppm as internal reference), acetone-$d_6$ (using 29.9 and 206.7 ppm for reference) at 75, 101 or 126 MHz. Infrared spectra were recorded using a Perkin Elmer Spectrum Two spectrometer using the attenuated total reflectance attachment (ATR). Wavenumbers in inverse centimeters ($cm^{-1}$) are reported for characteristic peaks. High-resolution mass spectra were obtained at SUNY Buffalo's mass spectrometry facility on Thermo Fisher Q- Exactive Liquid Chromatograph Orbitrap Tandem Mass Spectrometer. Melting points were obtained on an electrothermal melting point apparatus

and are reported uncorrected. 2-Aminopyridine and 8-hydroxyquinoline were purchased from Acros and used without further purification. 2-Chloro-3-cyanobenzaldehyde was purchased from Ambeed and used without further purification. α,α,α-Trifluorotolune was purchased froom Acros and distilled over $P_2O_5$. Reagent grade acetone and MeOH were used. Dichloromethane was dried through an alumina column using a solvent filtration system.

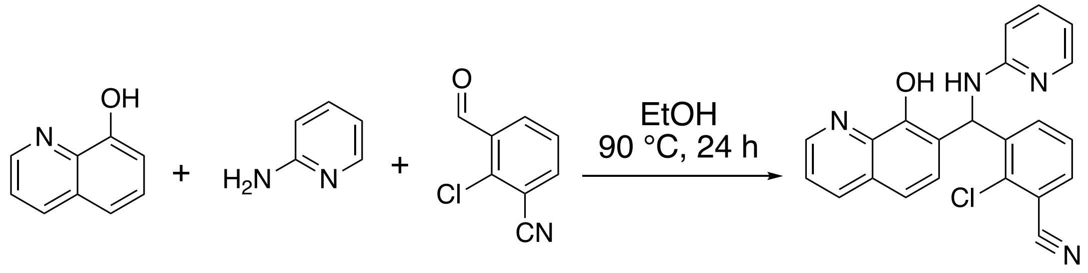


SC-62-1

**(±)-2-chloro-3-((8-hydroxyquinolin-7-yl)(pyridin-2-ylamino)methyl) benzonitrile (SC-62-1)** To a 250 mL round bottomed flask equipped with a magnetic stir bar, 2-chloro-3-cyanobenzaldehyde (1.0 g, 6.04 mmol, 1.0 equiv) was added along with 2-aminopyridine (568 mg, 6.04 mmol, 1.0 equiv). The solids were dissolved in 50 mL of absolute ethanol and the mixture was stirred until they fully dissolved, at which point 8-hydroxyquinoline (1.05 g, 7.25 mmol, 1.2 equiv) was added. The reaction flask was heated to 90 °C and refluxed for 24 h during which an off-white solid precipitated. The solution was allowed to cool to room temperature and to stand for several hours, then the resulting solid was isolated by filtration, giving **SC-62-1 (**1.75 g, 75% yield) as an off-white powder. Mp = 190-192 °C; $^{1}$H NMR (300 MHz, $CDCl_3$) δ 8.77 (d, *J* = 3.0 Hz, 1H), 8.13 (d, *J* = 7.2 Hz, 1H), 8.09 (d, *J* = 4.1 Hz, 1H), 7.97 (d, *J* = 7.9 Hz, 1H), 7.59 (d, *J* = 6.4 Hz, 1H), 7.50 – 7.26 (m, 6H), 6.70 (d, *J* = 6.4 Hz, 1H), 6.66 – 6.57 (m, 1H), 6.36 (d, *J* = 8.4 Hz, 1H), 5.55 (d, *J* = 6.2 Hz, 1H); $^{13}$C NMR (75 MHz, $CDCl_3$) δ 157.2, 149.8, 148.3, 141.5, 138.2, 137.7, 136.1, 136.0, 133.0, 132.9, 127.9, 127.4, 127.0, 122.2, 120.6, 117.9, 116.2, 114.3, 114.0, 107.1, 53.6; IR (neat film): 3346, 2923, 2233, 1600, 1571, 1517, 1501 cm$^{-1}$; HRMS (ESI) calculated for $C_{22}H_{16}ClN_4O$ $[M+H]^+$: 387.1016, found 387.1007.

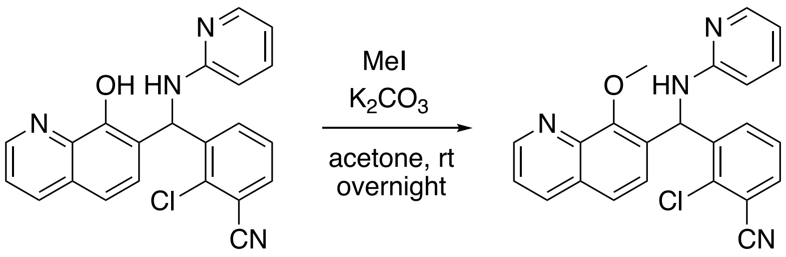

SC-62-1Me

**(±)-2-Chloro-3-((8-methoxyquinolin-7-yl)(pyridin-2-ylamino)methyl)benzonitrile (SC-62-1Me)**

To a 10 mL reaction tube, **SC-62-1** (50 mg, 0.129 mmol, 1 eq.) was added followed by 1.5 mL of acetone. MeI (1 M solution in acetone) was added to the reaction mixture (0.142 mL, 0.142 mmol, 1.1 eq.), followed by $K_2CO_3$ (35 mg, 0.259 mmol, 2 eq.). The reaction mixture was stirred at rt overnight. After ca. 16 h, 1.5 mL of deionized water was added, followed by 1.5 mL of $Et_2O$. The layers were separated and the aqueous layer was extracted with $Et_2O$. The combined organic layers were dried over $Na_2SO_4$, filtered and concentrated, yielding **SC-62-1-Me** in a crude mixture. Flash column chromatography on silica gel, 10-30% acetone:hexanes provided 35 mg of the pure methyl ether was isolated as a yellow solid in 68% yield. $^1$H NMR (400 MHz, $CDCl_3$) δ 8.92 (dd, *J* = 4.2, 1.7 Hz, 1H), 8.13 (dd, *J* = 8.3, 1.7 Hz, 1H), 8.08 – 8.02 (m, 1H), 7.83 (dd, *J* = 7.9, 1.6 Hz, 1H), 7.60 (dd, *J* = 7.7, 1.6 Hz, 1H), 7.53 (d, *J* = 8.5 Hz, 1H), 7.45 – 7.32 (m, 4H), 6.78 (d, *J* = 6.4 Hz, 1H), 6.65 – 6.57 (m, 1H), 6.36 (dd, *J* = 8.4, 1.0 Hz, 1H), 5.29 (dd, *J* = 6.7, 3.6 Hz, 1H), 4.05 (s, 3H). $^{13}$C NMR (101 MHz, $CDCl_3$) δ 157.1, 154.1, 149.7, 148.4, 142.8, 141.9, 137.7, 136.2, 136.1, 133.1, 133.0, 131.7, 129.7, 127.1, 126.4, 123.4, 121.6, 116.2, 114.5, 114.1, 107.1, 62.6, 53.5, 36.7, 29.7. HRMS (ESI) calculated for $C_{23}H_{18}ClN_4O$ $[M+H]^+$ : 401.1169; found 401.1163.

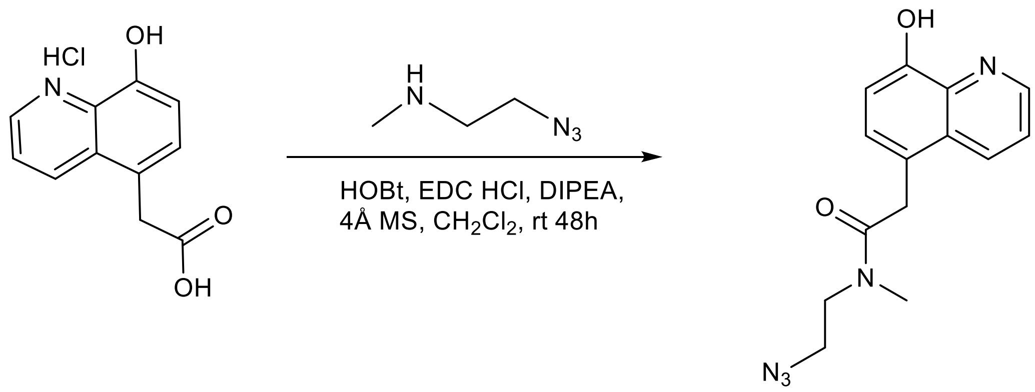


**N-(2-azidoethyl)-2-(8-hydroxyquinolin-5-yl)-N-methylacetamide (2)**

This procedure is modified from a previously reported coupling.[1] To a 100 mL round bottom flask equipped with a stir bar and flame-dried 4Å molecular sieves (200 mg), 5-(8-hydroxyquinolyl) acetic acid hydrochloride (**1**)[2] (100 mg, 0.42 mmol, 1 eq.) was added. This was followed by EDC•HCl (81 mg, 0.42 mmol, 1 eq.), anhydrous HOBt (57 mg, 0.42 mmol, 1 eq.), and 7.5 mL anhydrous $CH_2Cl_2$. The mixture was stirred at 0 °C for 15 minutes under argon. Diisopropyl ethyl amine (DIPEA, 0.15 mL, 0.86 mmol, 2.04 eq.) was added and the solution was stirred for 45 minutes at room temperature. 2-Azido-*N*-methylethan-1-amine[3] (21 mg, 0.21 mmol, 0.5 eq.) dissolved in 3.0 mL anhydrous $CH_2Cl_2$ was added and the solution was stirred for 48 hours at room temperature. The reaction mixture was washed with deionized $H_2O$ (3 x 50mL) and the organic layer was dried over $Na_2SO_4$. This layer was filtered and concentrated, then purified on a silica gel column using a 50-60% EtOAc/hexanes gradient. Like fractions resulting from the 55% mobile phase were combined and concentrated to yield 90 mg of amide **2** as a white solid (75% yield).

Mp = 103 °C; $^1$H NMR (400 MHz, $CDCl_3$) δ 8.79 (dd, $J$ = 4.2, 1.5 Hz, 1H), 8.37 (dd, $J$ = 8.5, 1.5 Hz, 1H), 7.48 (dd, $J$ = 8.5, 4.2 Hz, 1H), 7.29 (d, $J$ = 7.8 Hz 1H), 7.12 (d, $J$ = 7.8 Hz 1H), 4.04 (s, 2H), 3.60-3.46 (m, 4H), 3.18 (s, 3H); $^{13}$C NMR (101 MHz, $CDCl_3$) δ 171.2, 151.7, 147.6, 138.7, 133.1, 128.6, 127.7, 121.9, 121.3, 109.2, 49.5, 48.3, 37.9, 37.5; FTIR: 3317, 2933, 2123, 2093, 1637, 1579, 1508, 1477, 1416, 1398, 1277, 1190, 833, 782 cm$^{-1}$; HRMS (ESI) calculated for $C_{14}H_{16}N_5O_2$ [M+H]$^+$: 286.1301, found: 286.1298.

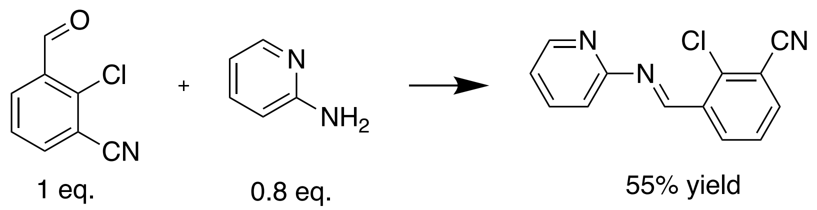


**(*E*)-2-chloro-3-((pyridin-2-ylimino)methyl)benzonitrile (3)**

3-Cyano-2-chlorobenzaldehyde (100 mg, 0.604 mmol, 1 eq.) was added to a 10 mL reaction tube, followed by 2-aminopyridine (45 mg, 0.406 mmol, 0.80 eq.) and 1.2 mL $PhCF_3$. Flame-dried activated 4Å molecular sieves (40 mg) were added to the reaction mixture. The reaction tube was capped then heated to 105 °C for 18 h. The reaction mixture was cooled to rt then diluted with $CH_2Cl_2$, filtered through Celite washing with $CH_2Cl_2$, then concentrated to yield the crude imine **B**. It was then immediately chromatographed on a silica gel column using 0-5% EtOAc : hexanes with 1% $Et_3N$, giving imine **3** (80 mg) as a white powder in 55% yield. Mp = 163 °C; $^1$H NMR (500 MHz, acetone-$d_6$) δ 9.77 (s, 1H), 8.64 (d, $J$ = 8.0 Hz, 1H), 8.57 (d, $J$ = 4.7 Hz, 1H), 8.11 (d, $J$ = 7.7 Hz, 1H), 7.96 (td, $J$ = 7.7, 2.0 Hz, 1H), 7.75 (t, $J$ = 7.8 Hz, 1H), 7.47 (d, $J$ = 7.9 Hz, 1H), 7.39 (dd, $J$ = 7.4, 4.7 Hz, 1H); $^{13}$C NMR (126 MHz, $CDCl_3$) δ 187.7, 157.6, 149.1, 140.1, 138.4, 136.5, 133.3, 133.0, 127.8, 127.4, 122.9, 120.5; HRMS (ESI) calculated for $C_{13}H_8ClN_3$ $[M]^+$: 242.0485, found 242.0505.

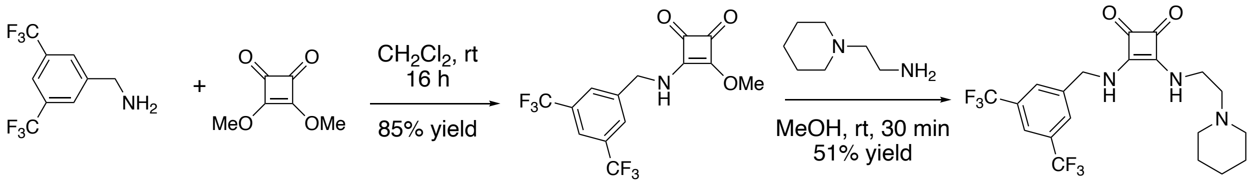


**3-((3,5-bis(trifluoromethyl)benzyl)amino)-4-((2-(piperidin-1- yl)ethyl)amino)cyclobut-3-ene-1,2-dione (4)**

The synthesis of squaramide **4** is adapted from a literature procedure.[4] To a dry 25 mL round bottom flask, 3,4-dimethoxycyclobut-3-ene-1,2-dione (142 mg, 1.0 mmol, 1.0 eq.) was added and dissolved in $CH_2Cl_2$ (4 mL). Separately, (3,5-bis(trifluoromethyl)phenyl)methanamine (255 mg,

1.05 mmol, 1.8 eq.) was dissolved in 1 mL $CH_2Cl_2$, then this solution was added to the round bottom flask. The reaction mixture was stirred at rt overnight, then filtered through Celite, washing with $CH_2Cl_2$. The filtrate was washed with 1M HCl then the organic layer was dried with $Na_2SO_4$. The organic layer was concentrated to yield 176 mg of 3-((3,5-bis(trifluoromethyl)benzyl)amino)-4-methoxycyclobut-3-ene-1,2-dione in an 85% yield.

3-((3,5-Bis(trifluoromethyl)benzyl)amino)-4-methoxycyclobut-3-ene-1,2-dione (176 mg, 0.500 mmol, 1 eq.) was dissolved in MeOH (4 mL) and added to a 25 mL round bottom flask. Separately, 2-(piperidin-1-yl)ethan-1-amine (53 mg, 0.413 mmol, 0.83 eq.) is dissolved in 1 mL MeOH, then added to the stirring reaction mixture in the round bottom flask. The product precipitated out of solution after 30 minutes and was filtered off, washing with cold MeOH, providing squaramide catalyst **4** (51% yield). Mp = 170 °C; $^1$H NMR (400 MHz, DMSO-d$_6$) δ 8.06 (d, *J* = 5.1 Hz, 3H), 7.50 (bs, 1H) 4.91 (d, *J* = 6.4 Hz, 2H), 3.61 (s, 1H), 2.41 (t, *J* = 6.2 Hz, 4H), 2.34 (s, 4H), 1.45 (s, 4H), 1.36 (d, *J* = 5.9 Hz, 3H); $^{13}$C NMR (101 MHz, DMSO-d$_6$) δ 182.2, 154.6, 143.5, 131.1, 130.8, 129.0, 124.8, 59.3, 54.5, 46.1, 25.9, 24.4; HRMS (ESI) calculated for $C_{20}H_{21}F_6N_3O_2$ $[M]^+$:450.1616, found 450.1588.

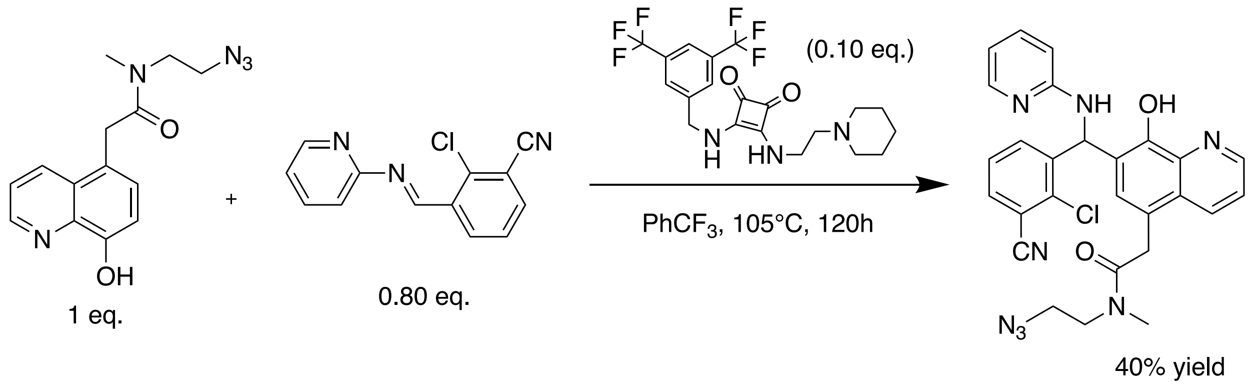


**(±)-*N*-(2-azidoethyl)-2-(7-((2-chloro-3-cyanophenyl)(pyridin-2-ylamino)methyl)-8-hydroxyquinolin-5-yl)-*N*-methylacetamide (SC-62-16)**

(Note: The 3-component Betti reaction did not work for this 5-substituted-8-hydroxy quinoline, it is much less reactive than 8-hydroxy quinoline.) To a 10 mL reaction tube, the imine **3** (42 mg,

0.175 mmol, 1 eq.) was added, followed by the amide **2** (40 mg, 0.14 mmol, 0.8 eq.) dissolved in $PhCF_3$ (0.5 mL). The squaramide catalyst **3** (8 mg, 0.04 mmol, 0.1 eq.) was also added to the reaction tube. Flame dried 4Å molecular sieves (30 mg) were added, and the reaction tube was sealed and kept in a dark environment at 105 °C for 5 days. The reaction mixture was cooled to rt, diluted with $CH_2Cl_2$, and filtered through Celite then concentrated to a crude mixture. The crude was then recrystallized out of MeOH to provide 42 mg of **SC-62-16** as light brown crystals in 40% yield. The NMR data indicates presence of amide rotamers due to hindered rotation. Mp = 162-164 °C; $^1$H NMR (400 MHz, $CDCl_3$) δ 8.79 (dd, *J* = 4.2, 1.5 Hz, 1H), 8.36 (ddd, *J* = 14.3, 8.6, 1.5 Hz, 1H), 8.08 (ddd, *J* = 5.0, 1.9, 0.8 Hz, 1H), 7.94 (dt, *J* = 7.9, 2.5 Hz, 1H), 7.59 (dd, *J* = 7.7, 1.6 Hz, 1H), 7.49 (ddd, *J* = 8.6, 4.2, 1.6 Hz, 1H), 7.45 – 7.30 (m, 2H), 7.25 (s, 1H), 6.69 (dd, *J* = 6.5, 2.4 Hz, 1H), 6.61 (ddd, *J* = 7.2, 5.0, 0.9 Hz, 1H), 6.38 (d, *J* = 8.4 Hz, 1H), 5.54 (d, *J* = 6.5 Hz, 1H), 4.08 – 3.91 (m, 2H), 3.51 (t, *J* = 5.4 Hz, 2H), 3.47 – 3.33 (m, 3H), 3.07 (s, 2H), 2.92 (s, 1H); $^{13}$C NMR (101 MHz, $CDCl_3$) δ 170.5, 157.8, 149.3, 148.0, 147.8, 142.9, 138.5, 136.8, 135.4, 133.7, 133.4, 132.8, 127.9, 127.5, 122.5, 121.7, 120.8, 115.9, 113.8, 113.0, 108.8, 51.8, 51.7, 49.2, 48.7, 48.5, 47.0, 37.2, 36.7, 35.8, 32.5; HRMS (ESI) calculated for $C_{27}H_{24}ClN_8O_2$ $[M+H]^+$: 527.1711, found 527.1704.

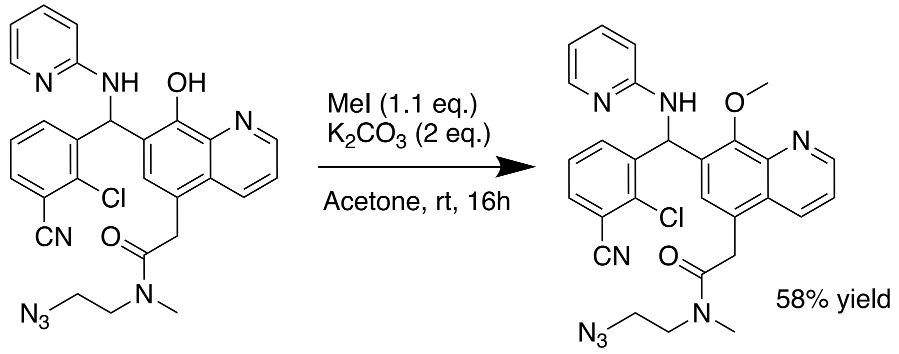


**(±)-*N*-(2-azidoethyl)-2-(7-((2-chloro-3-cyanophenyl)(pyridin-2-ylamino)methyl)-8-methoxyquinolin-5-yl)-*N*-methylacetamide (SC-62-16-Me)**

To a 10 mL reaction tube, **SC-62-16** (10 mg, 0.018 mmol, 1 eq.) was added then dissolved in 0.25 mL of acetone. MeI (1 M solution in acetone) was added to the reaction mixture (0.02 mL, 0.021 mmol, 1.1 eq.), followed by $K_2CO_3$ (5.25 mg, 0.038 mmol, 2 eq.). The reaction tube is left to stir at rt overnight (ca. 16 h), then 1 mL of deionized water was added, followed by 1 mL $Et_2O$. The $Et_2O$ layer was extracted and concentrated to give **SC-62-16-Me** in a crude mixture that was then

subject to flash column chromatography on silica gel, 30-60% acetone : hexanes. The pure methyl ether (6 mg) was isolated as a white solid in 58% yield. NMR data indicate presence of amide rotamers due to hindered rotation. $^1$H NMR (400 MHz, $CDCl_3$) δ 8.93 (dd, *J* = 4.1, 1.6 Hz, 1H), 8.31 (ddd, *J* = 16.7, 8.6, 1.6 Hz, 1H), 8.07 (d, *J* = 5.0 Hz, 1H), 7.80 (dt, *J* = 7.9, 2.2 Hz, 1H), 7.61 (dd, *J* = 7.7, 1.6 Hz, 1H), 7.49 – 7.31 (m, 4H), 7.25 (s, 1H), 6.79 (dd, *J* = 6.4, 2.4 Hz, 1H), 6.66 – 6.58 (m, 1H), 6.37 (d, *J* = 8.3 Hz, 1H), 5.26 (s, 1H), 4.01 (d, *J* = 5.9 Hz, 4H), 3.51 (dd, *J* = 6.1, 4.4 Hz, 2H), 3.47 – 3.42 (m, 2H), 3.42 – 3.32 (m, 2H), 3.05 (s, 2H), 2.92 (s, 1H); $^{13}$C NMR (101 MHz, $CDCl_3$) δ 170.69, 170.65, 157.1, 153.5, 149.5, 149.4, 148.18, 143.15, 141.9, 137.7, 136.1, 133.2, 133.0, 132.9, 131.1, 128.71, 128.65, 127.4, 127.3, 127.2, 127.1, 127.0, 121.6, 121.5, 116.2, 114.4, 114.03, 113.98, 107.42, 107.35, 62.58, 62.56, 53.38, 53.30, 49.23, 48.91, 48.13, 38.1, 37.3, 33.4, 29.7; HRMS (ESI) calculated for $C_{28}H_{25}ClN_8O_2$ $[M]^+$ 541.1687, found 541.1905.

**References**

(1) Zhang, M.; Gallagher, J. A.; Coppock, M. B.; Pantzar, L. M.; Williams, M. E., Cooperative Assembly of Zn Cross-Linked Artificial Tripeptides with Pendant Hydroxyquinoline Ligands. *Inorg. Chem.* **2012,** *51*, 11315-11323.

(2) Warner, V. D.; Sane, J. N.; Mirth, D. B., Synthesis and in Vitro Evaluation of 8-Hydroxyquinoline Analogs as Inhibitors of Dental Plaque. *J. Med. Chem.* **1976,** *19*, 167-169.

(3) Xiao, C.; Cheng, Y.; Zhang, Y.; Ding, J.; He, C.; Zhuang, X.; Chen, X., Side Chain Impacts on pH- and Thermo-Responsiveness of Tertiary Amine Functionalized Polypeptides. *J. Polymer Sci. Part A* **2014,** *52*, 671-679.

(4) Malerich, J. P.; Hagihara, K.; Rawal, V. H., Chiral Squaramide Derivatives are Excellent Hydrogen Bond Donor Catalysts. *J. Am. Chem. Soc.* **2008,** *130*, 14416-14417.

## (±)-2-chloro-3-((8-hydroxyquinolin-7-yl)(pyridin-2-ylamino)methyl) benzonitrile (SC-62-1)

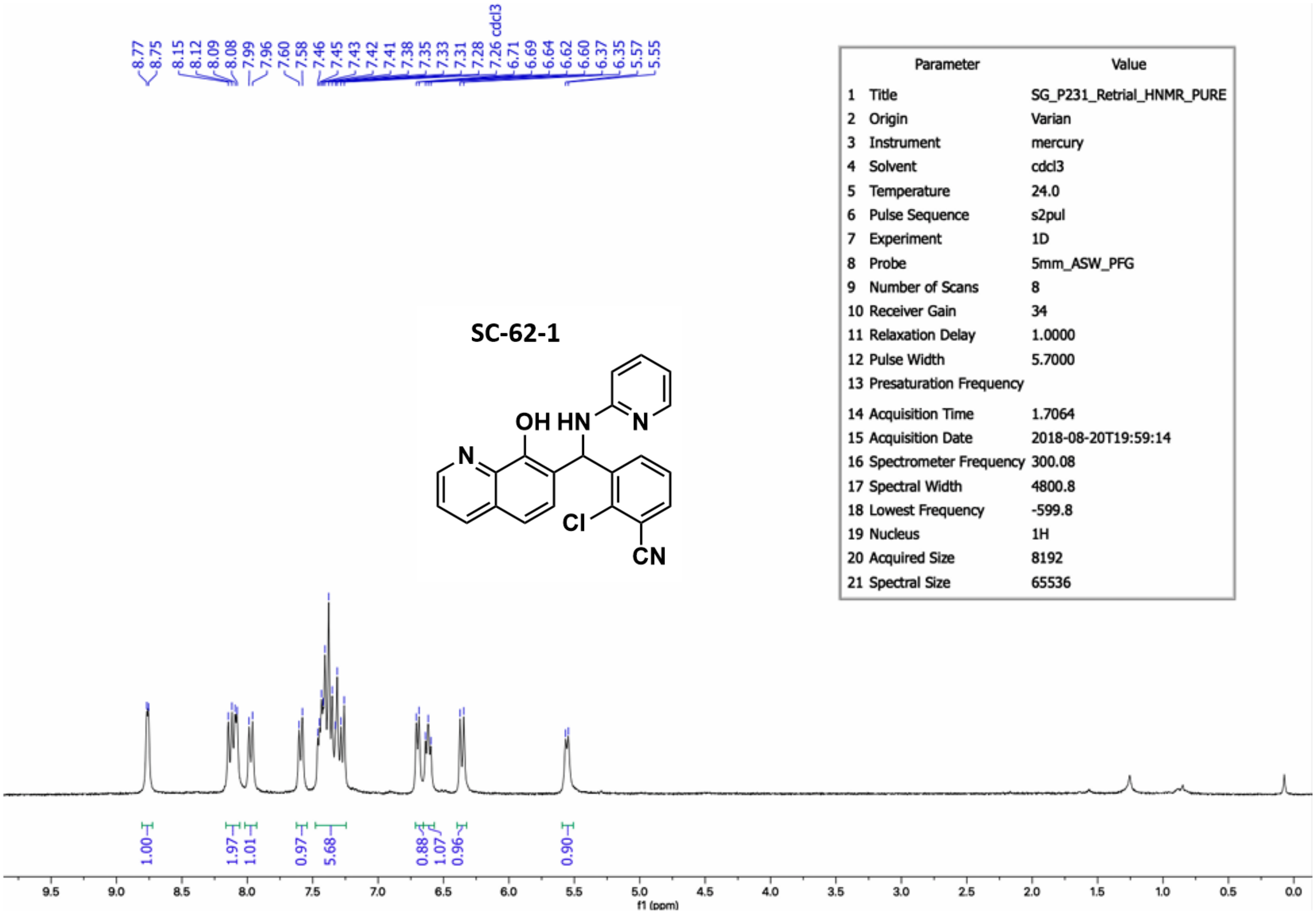


| | Parameter | Value |
|---|---|---|
| 1 | Title | SG_P231_Retrial_HNMR_PURE |
| 2 | Origin | Varian |
| 3 | Instrument | mercury |
| 4 | Solvent | cdcl3 |
| 5 | Temperature | 24.0 |
| 6 | Pulse Sequence | s2pul |
| 7 | Experiment | 1D |
| 8 | Probe | 5mm_ASW_PFG |
| 9 | Number of Scans | 8 |
| 10 | Receiver Gain | 34 |
| 11 | Relaxation Delay | 1.0000 |
| 12 | Pulse Width | 5.7000 |
| 13 | Presaturation Frequency | |
| 14 | Acquisition Time | 1.7064 |
| 15 | Acquisition Date | 2018-08-20T19:59:14 |
| 16 | Spectrometer Frequency | 300.08 |
| 17 | Spectral Width | 4800.8 |
| 18 | Lowest Frequency | -599.8 |
| 19 | Nucleus | 1H |
| 20 | Acquired Size | 8192 |
| 21 | Spectral Size | 65536 |

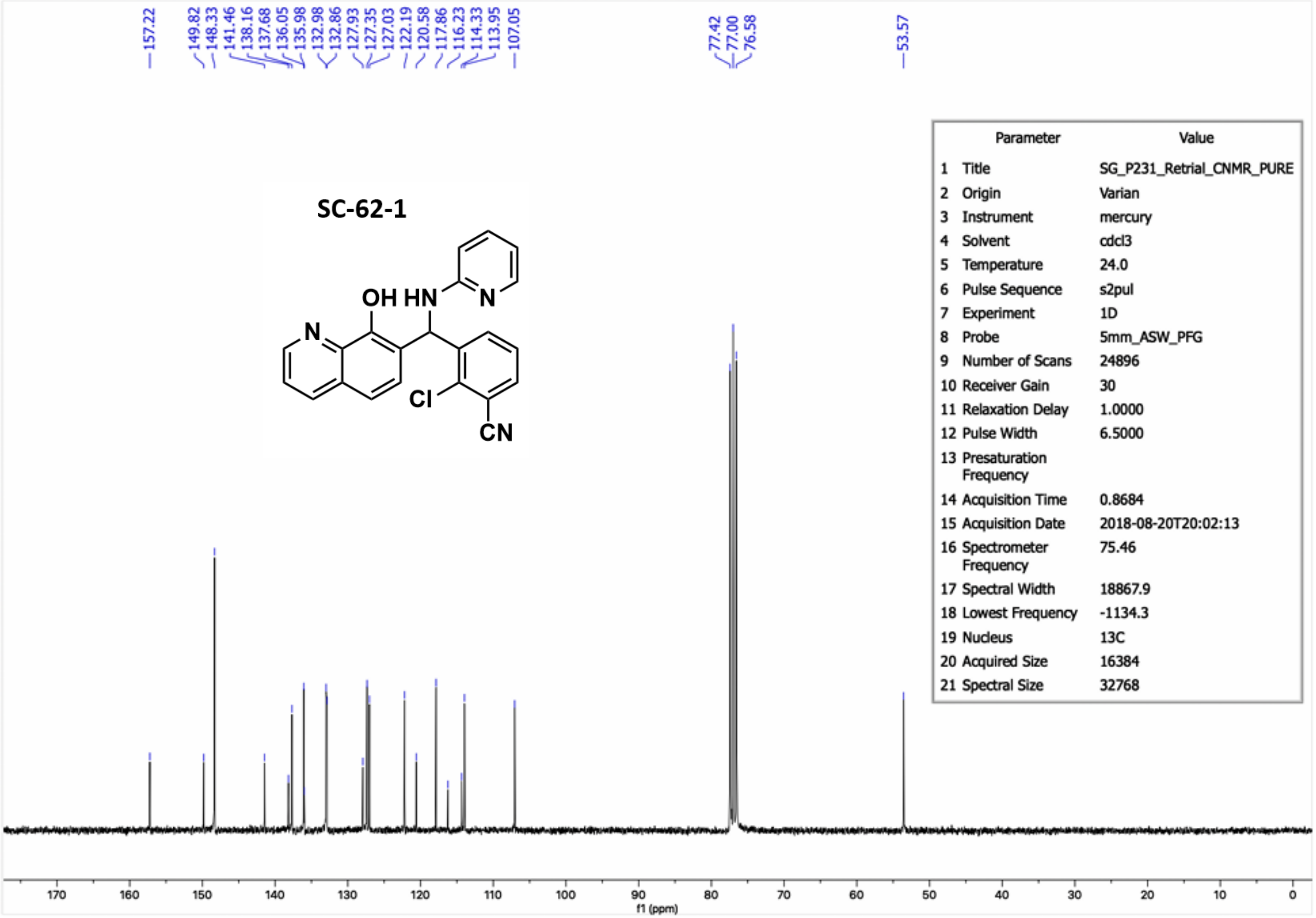


| | Parameter | Value |
|---|---|---|
| 1 | Title | SG_P231_Retrial_CNMR_PURE |
| 2 | Origin | Varian |
| 3 | Instrument | mercury |
| 4 | Solvent | cdcl3 |
| 5 | Temperature | 24.0 |
| 6 | Pulse Sequence | s2pul |
| 7 | Experiment | 1D |
| 8 | Probe | 5mm_ASW_PFG |
| 9 | Number of Scans | 24896 |
| 10 | Receiver Gain | 30 |
| 11 | Relaxation Delay | 1.0000 |
| 12 | Pulse Width | 6.5000 |
| 13 | Presaturation Frequency | |
| 14 | Acquisition Time | 0.8684 |
| 15 | Acquisition Date | 2018-08-20T20:02:13 |
| 16 | Spectrometer Frequency | 75.46 |
| 17 | Spectral Width | 18867.9 |
| 18 | Lowest Frequency | -1134.3 |
| 19 | Nucleus | 13C |
| 20 | Acquired Size | 16384 |
| 21 | Spectral Size | 32768 |

**2-chloro-3-((8-methoxyquinolin-7-yl)(pyridin-2-ylamino)methyl)benzonitrile (SC-62-1Me)**

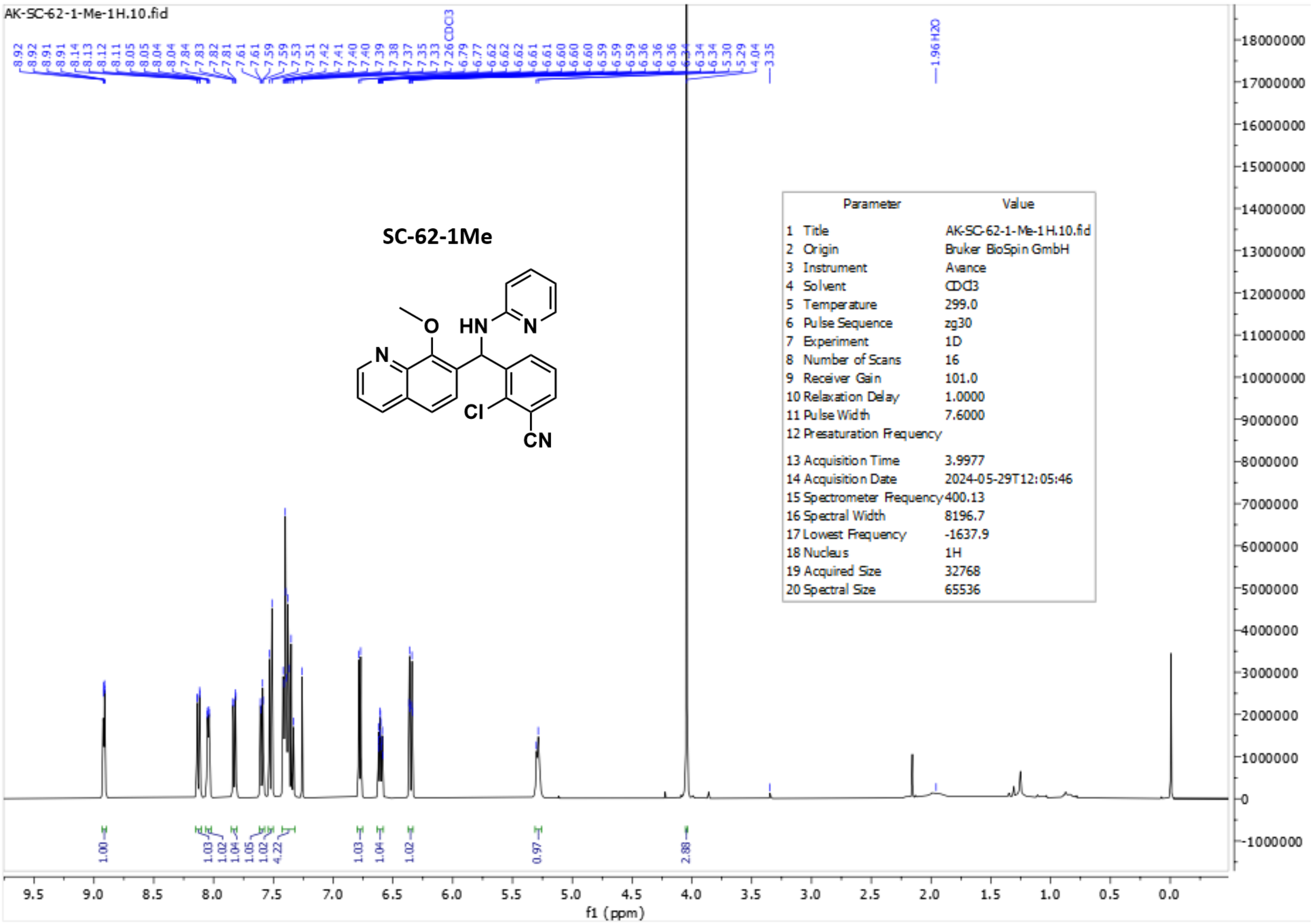

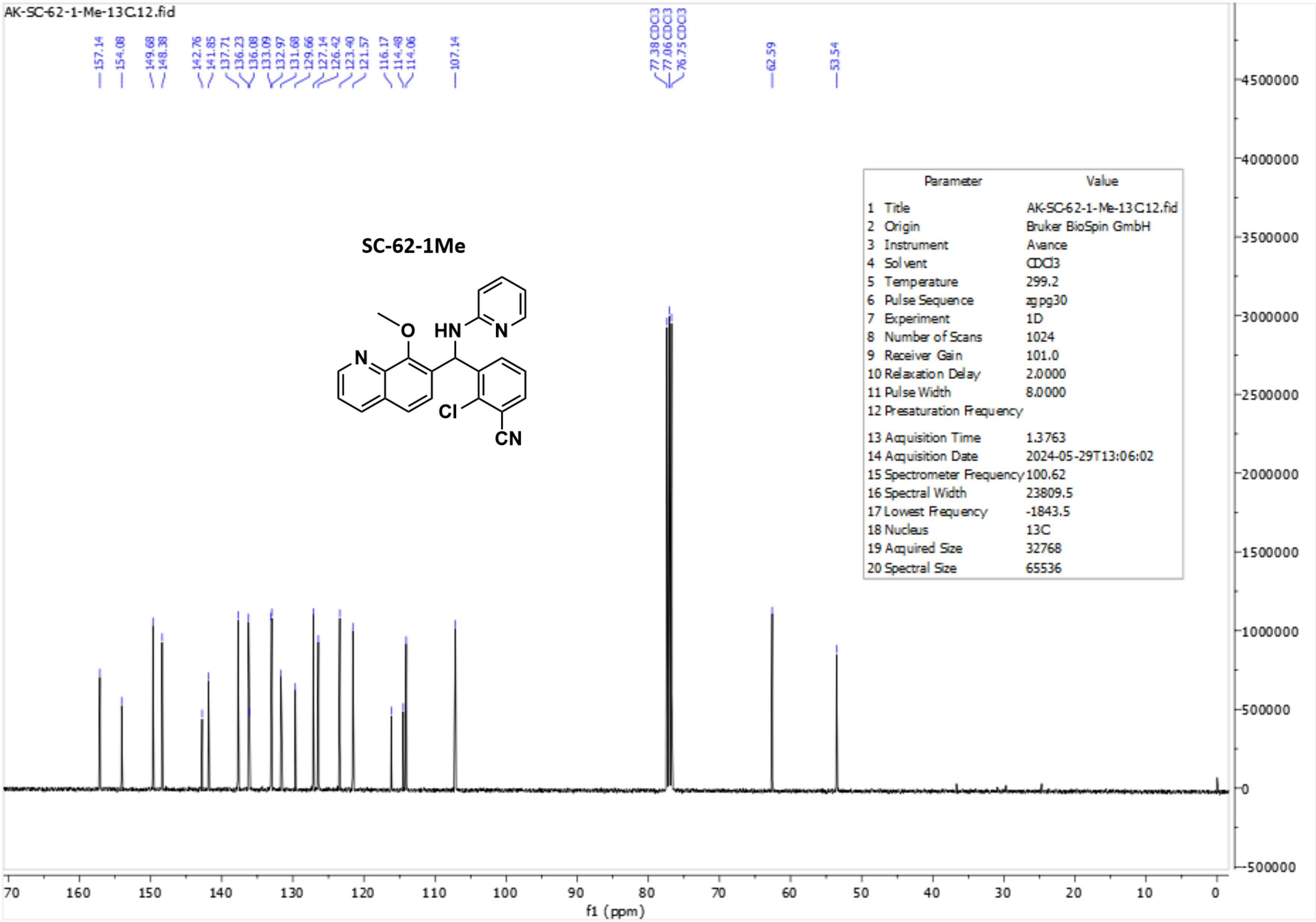


| Parameter | Value |
|---|---|
| 1 Title | AK-SC-62-1-Me-13C.12.fid |
| 2 Origin | Bruker BioSpin GmbH |
| 3 Instrument | Avance |
| 4 Solvent | CDCl3 |
| 5 Temperature | 299.2 |
| 6 Pulse Sequence | zgpg30 |
| 7 Experiment | 1D |
| 8 Number of Scans | 1024 |
| 9 Receiver Gain | 101.0 |
| 10 Relaxation Delay | 2.0000 |
| 11 Pulse Width | 8.0000 |
| 12 Presaturation Frequency | |
| 13 Acquisition Time | 1.3763 |
| 14 Acquisition Date | 2024-05-29T13:06:02 |
| 15 Spectrometer Frequency | 100.62 |
| 16 Spectral Width | 23809.5 |
| 17 Lowest Frequency | -1843.5 |
| 18 Nucleus | 13C |
| 19 Acquired Size | 32768 |
| 20 Spectral Size | 65536 |

**N-(2-azidoethyl)-2-(8-hydroxyquinolin-5-yl)-N-methylacetamide (2)**

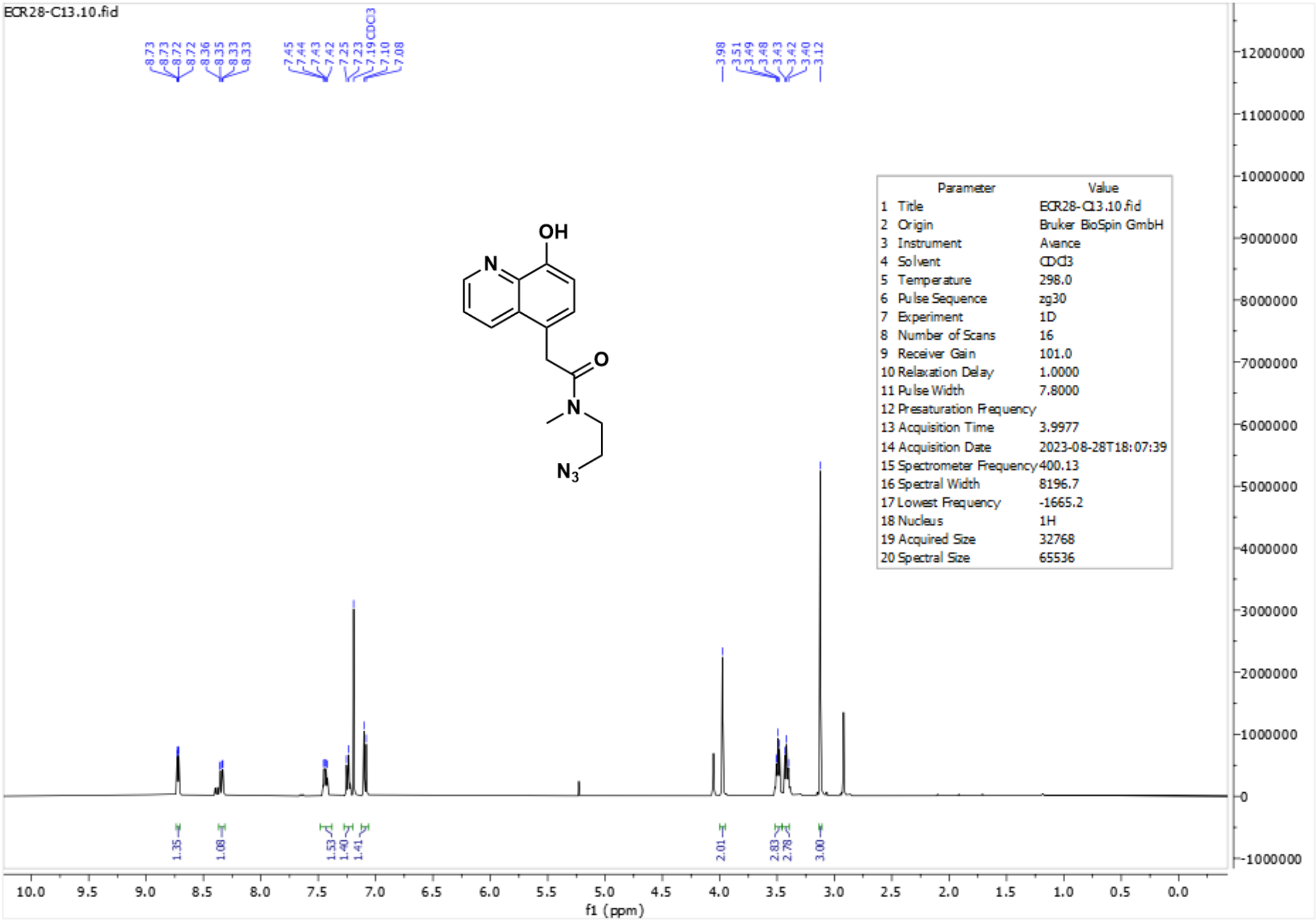

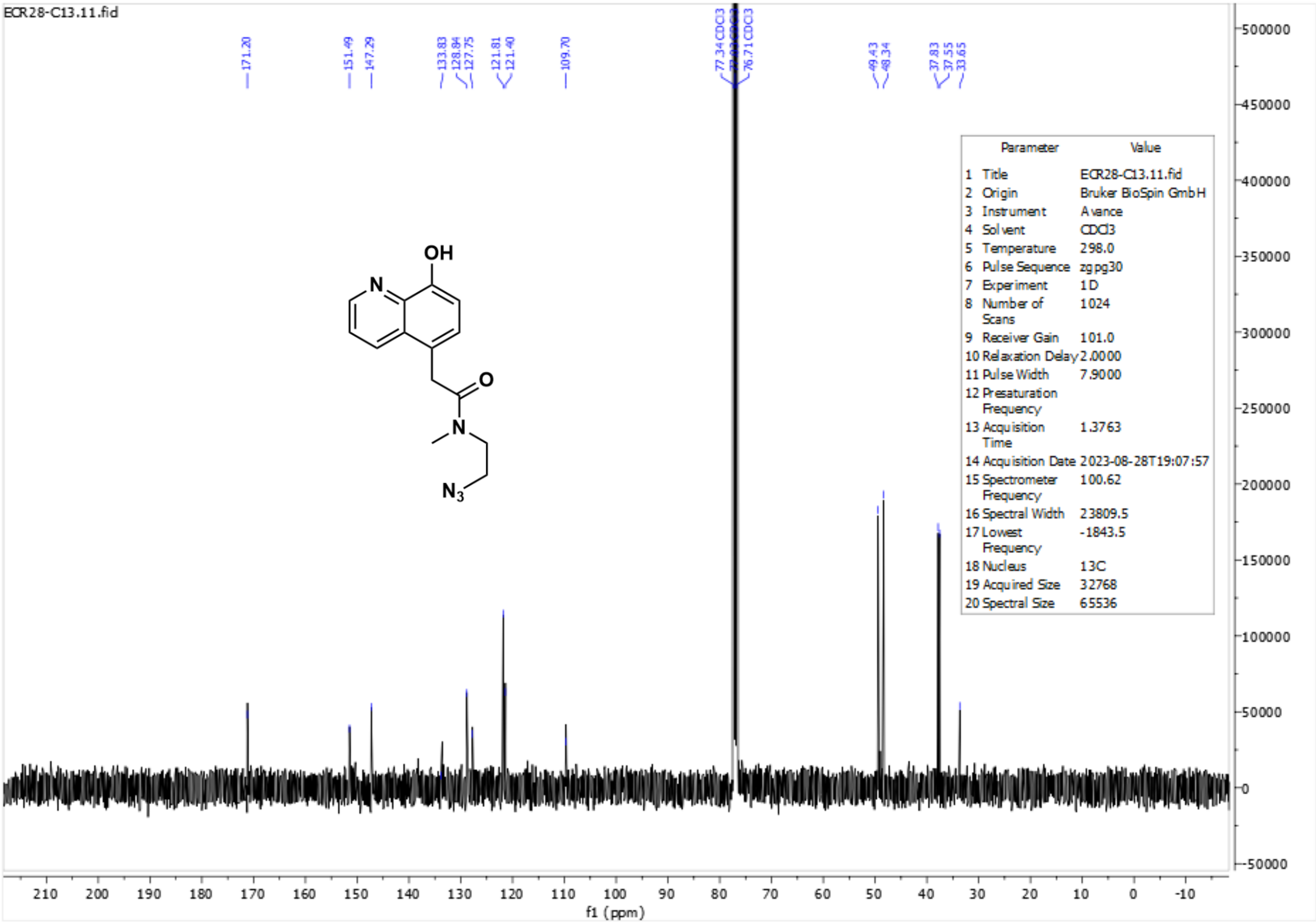
ECR28-C13.11.fid
171.20
151.49
147.29
133.83
128.84
127.75
121.81
121.40
109.70
77.34 CDCl3
76.71 CDCl3
49.43
48.34
37.83
37.55
33.65
OH
N
O
N
N3
Parameter | Value
1 Title | ECR28-C13.11.fid
2 Origin | Bruker BioSpin GmbH
3 Instrument | Avance
4 Solvent | CDCl3
5 Temperature | 298.0
6 Pulse Sequence | zgpg30
7 Experiment | 1D
8 Number of Scans | 1024
9 Receiver Gain | 101.0
10 Relaxation Delay | 2.0000
11 Pulse Width | 7.9000
12 Presaturation Frequency
13 Acquisition Time | 1.3763
14 Acquisition Date | 2023-08-28T19:07:57
15 Spectrometer Frequency | 100.62
16 Spectral Width | 23809.5
17 Lowest Frequency | -1843.5
18 Nucleus | 13C
19 Acquired Size | 32768
20 Spectral Size | 65536
f1 (ppm)

**(E)-2-chloro-3-((pyridin-2-ylimino)methyl)benzonitrile (3)**

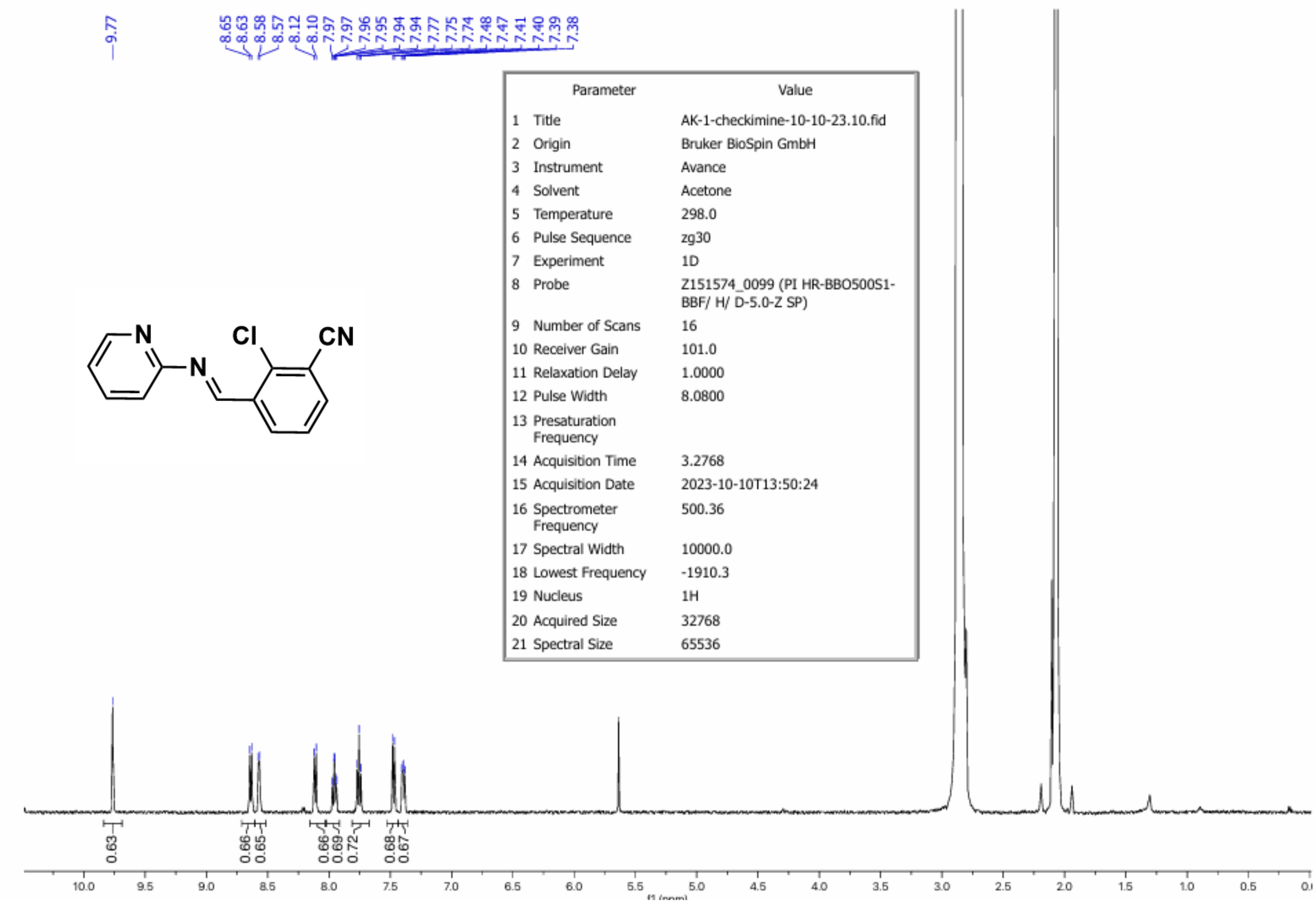


| | Parameter | Value |
|---|---|---|
| 1 | Title | AK-1-checkimine-10-10-23.10.fid |
| 2 | Origin | Bruker BioSpin GmbH |
| 3 | Instrument | Avance |
| 4 | Solvent | Acetone |
| 5 | Temperature | 298.0 |
| 6 | Pulse Sequence | zg30 |
| 7 | Experiment | 1D |
| 8 | Probe | Z151574_0099 (PI HR-BBO500S1-BBF/ H/ D-5.0-Z SP) |
| 9 | Number of Scans | 16 |
| 10 | Receiver Gain | 101.0 |
| 11 | Relaxation Delay | 1.0000 |
| 12 | Pulse Width | 8.0800 |
| 13 | Presaturation Frequency | |
| 14 | Acquisition Time | 3.2768 |
| 15 | Acquisition Date | 2023-10-10T13:50:24 |
| 16 | Spectrometer Frequency | 500.36 |
| 17 | Spectral Width | 10000.0 |
| 18 | Lowest Frequency | -1910.3 |
| 19 | Nucleus | 1H |
| 20 | Acquired Size | 32768 |
| 21 | Spectral Size | 65536 |

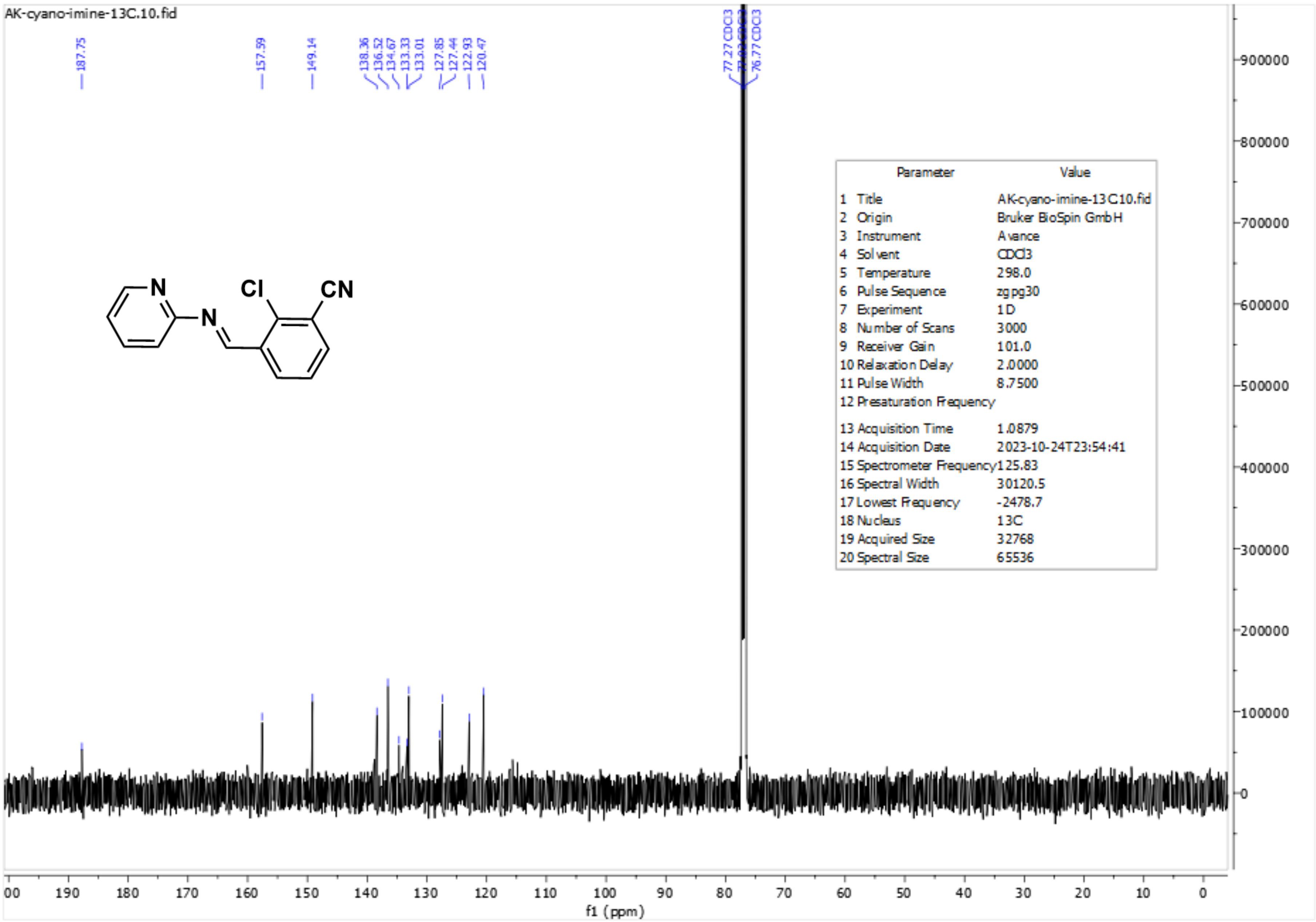


| Parameter | Value |
|---|---|
| 1 Title | AK-cyano-imine-13C10.fid |
| 2 Origin | Bruker BioSpin GmbH |
| 3 Instrument | Avance |
| 4 Solvent | CDCl3 |
| 5 Temperature | 298.0 |
| 6 Pulse Sequence | zgpg30 |
| 7 Experiment | 1D |
| 8 Number of Scans | 3000 |
| 9 Receiver Gain | 101.0 |
| 10 Relaxation Delay | 2.0000 |
| 11 Pulse Width | 8.7500 |
| 12 Presaturation Frequency | |
| 13 Acquisition Time | 1.0879 |
| 14 Acquisition Date | 2023-10-24T23:54:41 |
| 15 Spectrometer Frequency | 125.83 |
| 16 Spectral Width | 30120.5 |
| 17 Lowest Frequency | -2478.7 |
| 18 Nucleus | 13C |
| 19 Acquired Size | 32768 |
| 20 Spectral Size | 65536 |

**3-((3,5-bis(trifluoromethyl)benzyl)amino)-4-((2-(piperidin-1- yl)ethyl)amino)cyclobut-3-ene-1,2-dione (4)**

| | Parameter | Value |
|---|---|---|
| 1 | Title | AK-achiral-squaramide-1H.10.fid |
| 2 | Origin | Bruker BioSpin GmbH |
| 3 | Instrument | Avance |
| 4 | Solvent | DMSO |
| 5 | Temperature | 299.0 |
| 6 | Pulse Sequence | zg30 |
| 7 | Experiment | 1D |
| 8 | Probe | Z163739_0188 (PI HR-BBO400S1-BBF/H/ D-5.0-Z SP) |
| 9 | Number of Scans | 16 |
| 10 | Receiver Gain | 101.0 |
| 11 | Relaxation Delay | 1.0000 |
| 12 | Pulse Width | 7.6000 |
| 13 | Presaturation Frequency | |
| 14 | Acquisition Time | 3.9977 |
| 15 | Acquisition Date | 2024-06-11T15:14:33 |
| 16 | Spectrometer Frequency | 400.13 |
| 17 | Spectral Width | 8196.7 |
| 18 | Lowest Frequency | -1627.6 |
| 19 | Nucleus | 1H |
| 20 | Acquired Size | 32768 |
| 21 | Spectral Size | 65536 |

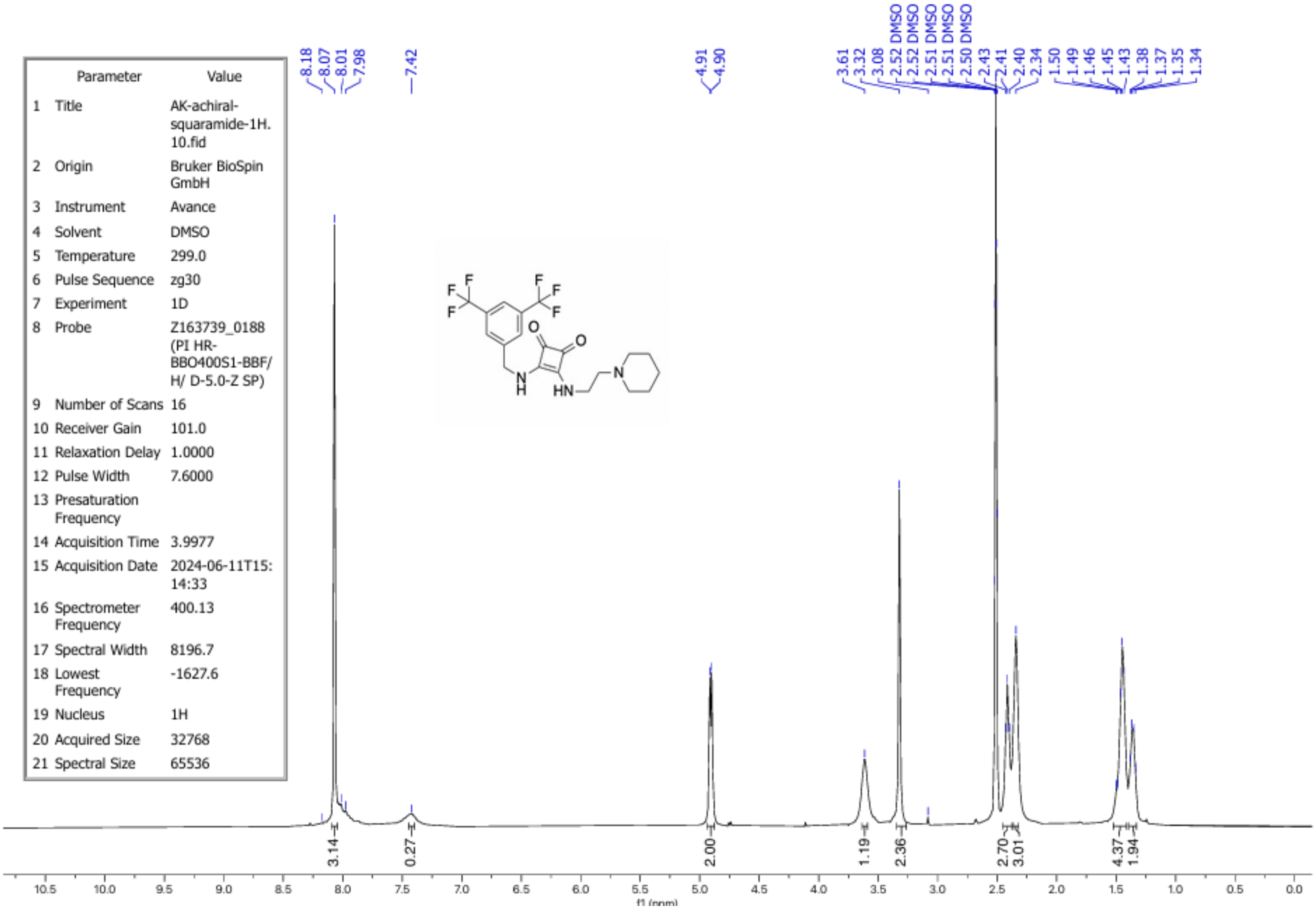

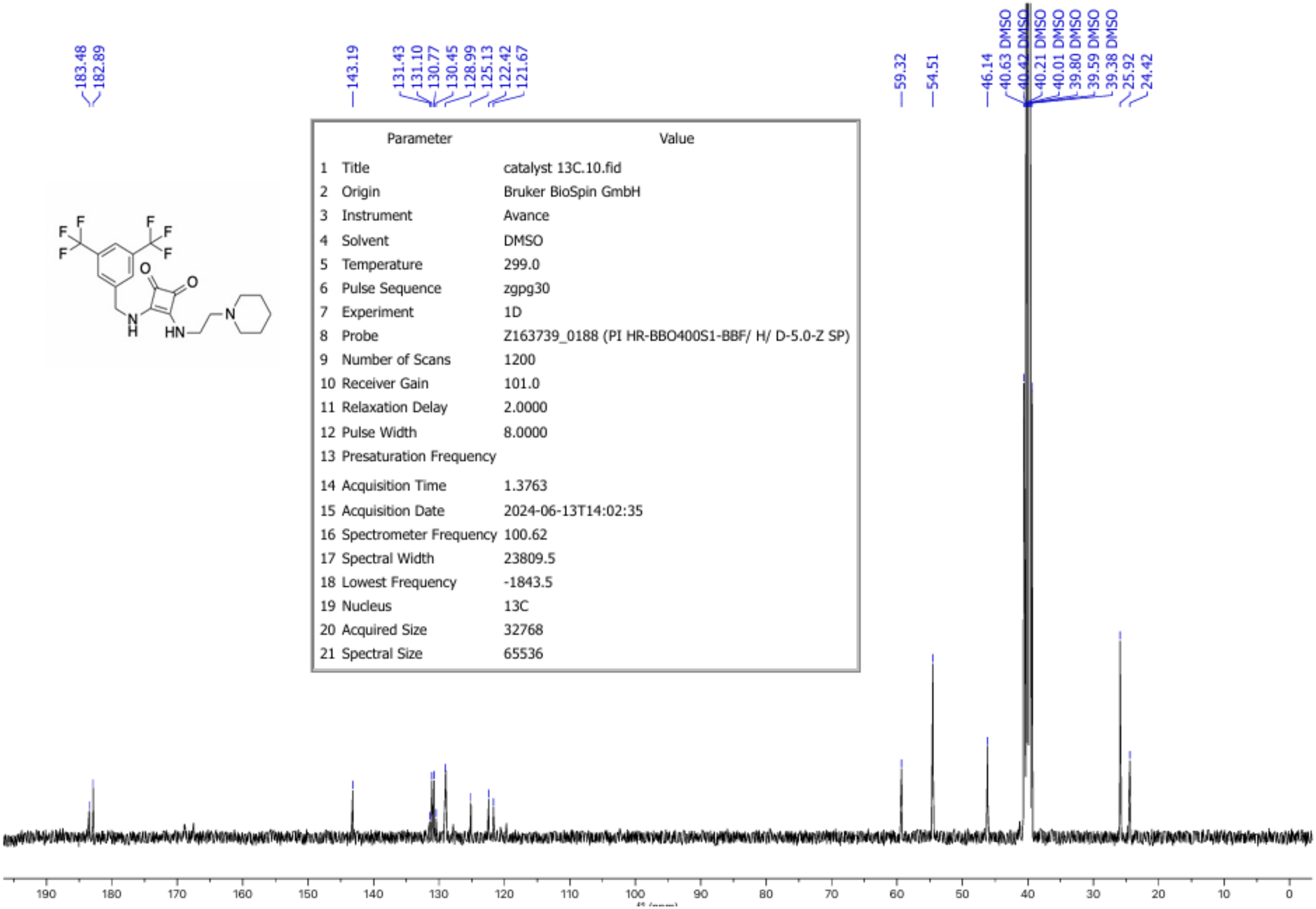


| | Parameter | Value |
|---|---|---|
| 1 | Title | catalyst 13C.10.fid |
| 2 | Origin | Bruker BioSpin GmbH |
| 3 | Instrument | Avance |
| 4 | Solvent | DMSO |
| 5 | Temperature | 299.0 |
| 6 | Pulse Sequence | zgpg30 |
| 7 | Experiment | 1D |
| 8 | Probe | Z163739_0188 (PI HR-BBO400S1-BBF/ H/ D-5.0-Z SP) |
| 9 | Number of Scans | 1200 |
| 10 | Receiver Gain | 101.0 |
| 11 | Relaxation Delay | 2.0000 |
| 12 | Pulse Width | 8.0000 |
| 13 | Presaturation Frequency | |
| 14 | Acquisition Time | 1.3763 |
| 15 | Acquisition Date | 2024-06-13T14:02:35 |
| 16 | Spectrometer Frequency | 100.62 |
| 17 | Spectral Width | 23809.5 |
| 18 | Lowest Frequency | -1843.5 |
| 19 | Nucleus | 13C |
| 20 | Acquired Size | 32768 |
| 21 | Spectral Size | 65536 |

**N-(2-azidoethyl)-2-(7-((2-chloro-3-cyanophenyl)(pyridin-2-ylamino)methyl)-8 hydroxyquinolin-5-yl)-N-methylacetamide (SC-62-16)**

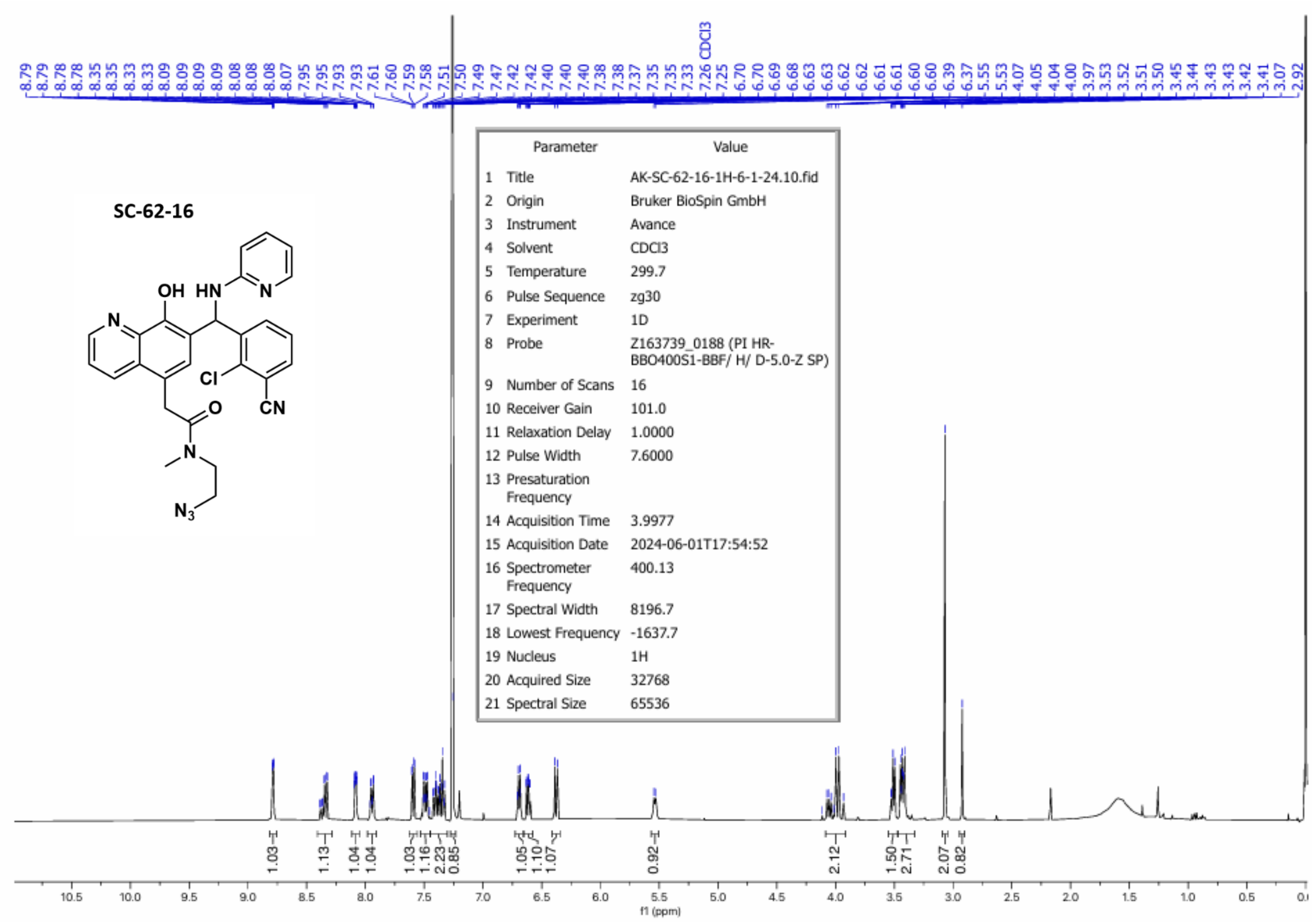

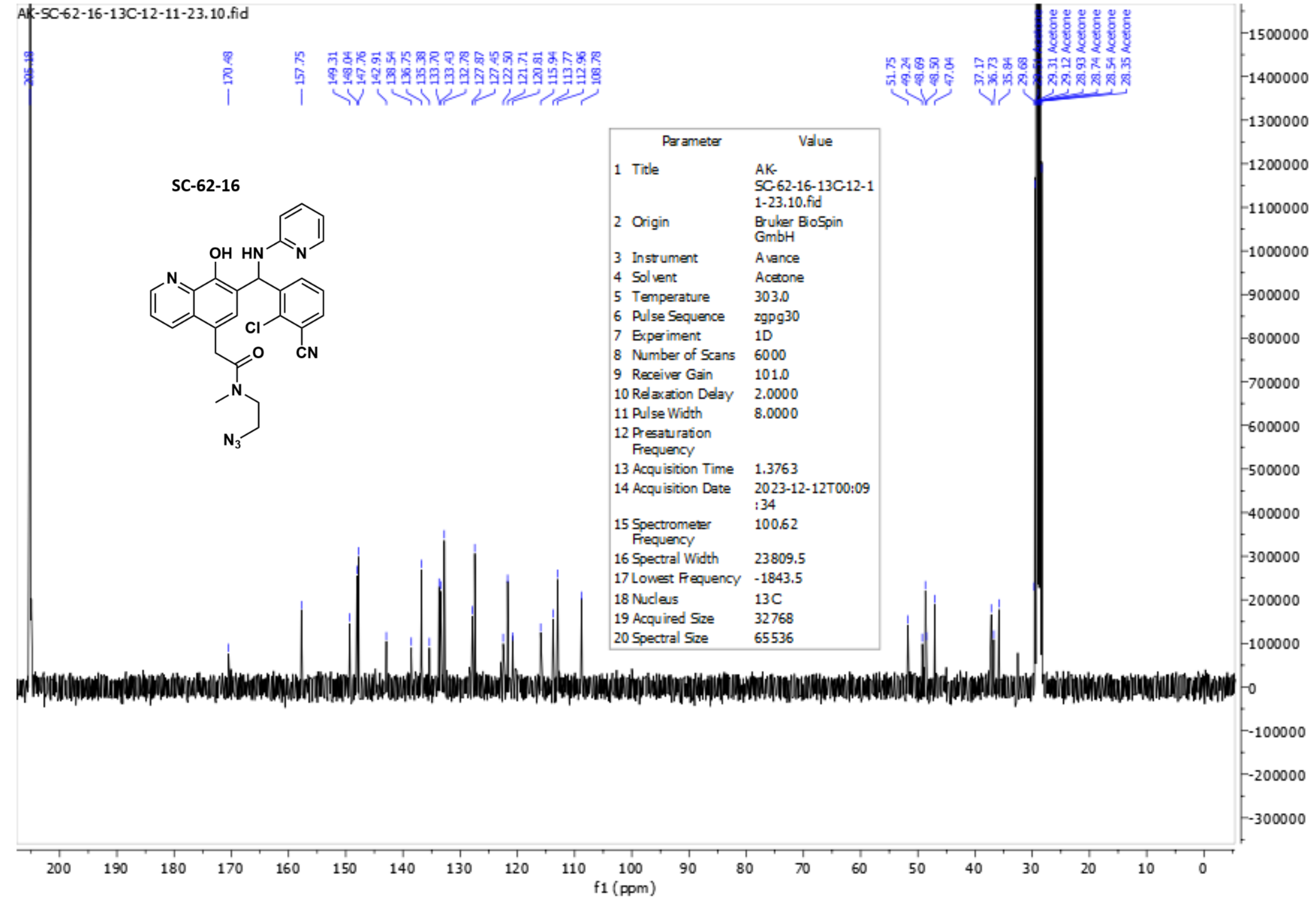


| | Parameter | Value |
|---|---|---|
| 1 | Title | AK-SC-62-16-13C-12-11-23.10.fid |
| 2 | Origin | Bruker BioSpin GmbH |
| 3 | Instrument | Avance |
| 4 | Solvent | Acetone |
| 5 | Temperature | 303.0 |
| 6 | Pulse Sequence | zgpg30 |
| 7 | Experiment | 1D |
| 8 | Number of Scans | 6000 |
| 9 | Receiver Gain | 101.0 |
| 10 | Relaxation Delay | 2.0000 |
| 11 | Pulse Width | 8.0000 |
| 12 | Presaturation Frequency | |
| 13 | Acquisition Time | 1.3763 |
| 14 | Acquisition Date | 2023-12-12T00:09:34 |
| 15 | Spectrometer Frequency | 100.62 |
| 16 | Spectral Width | 23809.5 |
| 17 | Lowest Frequency | -1843.5 |
| 18 | Nucleus | 13C |
| 19 | Acquired Size | 32768 |
| 20 | Spectral Size | 65536 |

**N-(2-azidoethyl)-2-(7-((2-chloro-3-cyanophenyl)(pyridin-2-ylamino)methyl)-8 methoxyquinolin-5-yl)-N-methylacetamide (SC-62-16-Me)**

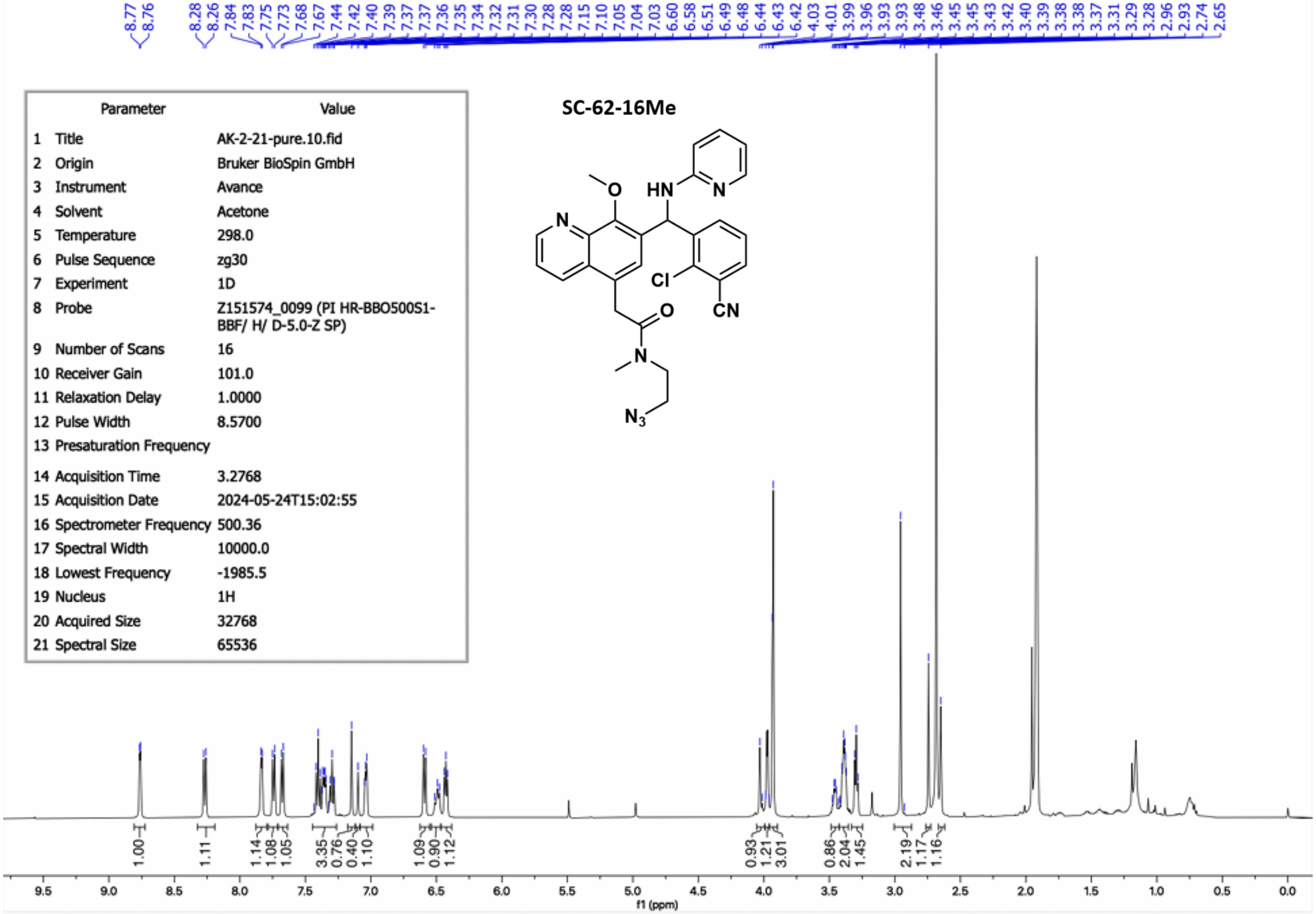

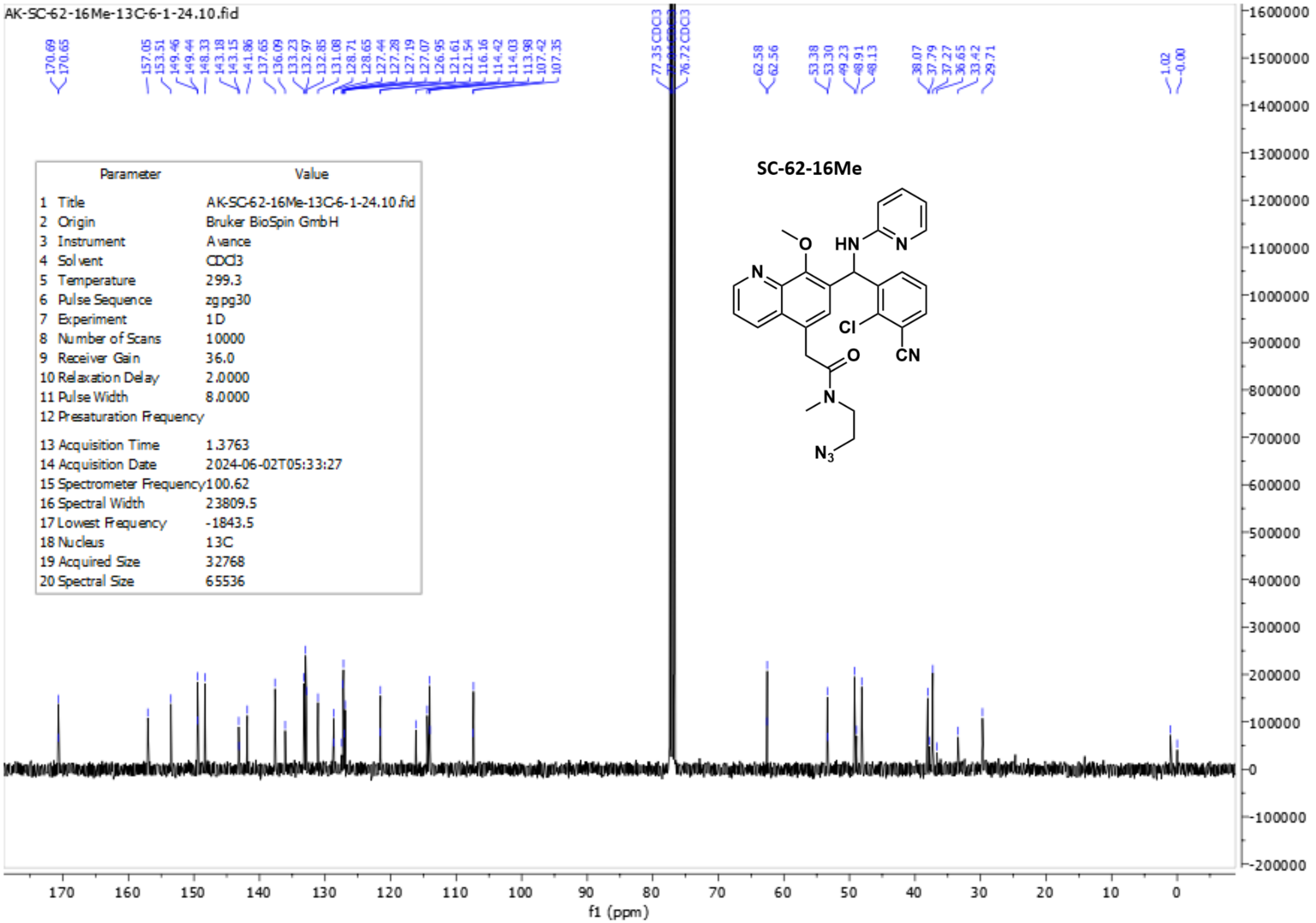
AK-SC-62-16Me-13C-6-1-24.10.fid
170.69
170.65
157.05
153.51
149.46
149.44
148.33
143.18
143.15
141.86
137.65
136.09
133.23
132.97
132.85
131.08
128.71
128.65
127.44
127.28
127.19
127.07
126.95
121.61
121.54
116.16
114.42
114.03
113.98
107.42
107.35
77.35 CDCl3
76.72 CDCl3
62.58
62.56
53.38
53.30
49.23
48.91
48.13
38.07
37.79
37.27
36.65
33.42
29.71
1.02
-0.00
Parameter Value
1 Title AK-SC-62-16Me-13C-6-1-24.10.fid
2 Origin Bruker BioSpin GmbH
3 Instrument Avance
4 Solvent CDCl3
5 Temperature 299.3
6 Pulse Sequence zgpg30
7 Experiment 1D
8 Number of Scans 10000
9 Receiver Gain 36.0
10 Relaxation Delay 2.0000
11 Pulse Width 8.0000
12 Presaturation Frequency
13 Acquisition Time 1.3763
14 Acquisition Date 2024-06-02T05:33:27
15 Spectrometer Frequency 100.62
16 Spectral Width 23809.5
17 Lowest Frequency -1843.5
18 Nucleus 13C
19 Acquired Size 32768
20 Spectral Size 65536
SC-62-16Me
O
HN
N
N
Cl
CN
O
N
N3
f1 (ppm)
